# Theoretical in-Solution Conformational/Tautomeric Analyses for Chain Systems with Conjugated Double Bonds Involving Nitrogen(s)

**DOI:** 10.3390/ijms160510767

**Published:** 2015-05-13

**Authors:** Peter I. Nagy

**Affiliations:** Center for Drug Design and Development, the University of Toledo, Toledo, OH 43606, USA; E-Mail: pnagy@utnet.utoledo.edu; Tel.: +1-419-530-2167; Fax: +1-419-530-7946

**Keywords:** *s-cis*/*s-trans* equilibrium, IEF-PCM/B97D/aug-cc-pvqz, IEF-PCM/CCSD(T)/CBS, FEP/MC, tautomerization mechanism

## Abstract

Conformational/tautomeric transformations for X=CH–CH=Y structures (X = CH_2_, O, NH and Y = NH) have been studied in the gas phase, in dichloromethane and in aqueous solutions. The paper is a continuation of a former study where *s-cis*/*s-trans* conformational equilibria were predicted for analogues. The *s-trans* conformation is preferred for the present molecules in the gas phase on the basis of its lowest internal free energy as calculated at the B97D/aug-cc-pvqz and CCSD(T)_CBS_ (coupled-cluster singles and doubles with non-iterative triples extrapolated to the complete basis set) levels. Transition state barriers are of 29–36 kJ/mol for rotations about the central C–C bonds. In solution, an *s-trans* form is still favored on the basis of its considerably lower internal free energy compared with the *s-cis* forms as calculated by IEF-PCM (integral-equation formalism of the polarizable continuum dielectric solvent model) at the theoretical levels indicated. A tetrahydrate model in the supermolecule/continuum approach helped explore the 2solute-solvent hydrogen bond pattern. The calculated transition state barrier for rotation about the C–C bond decreased to 27 kJ/mol for the tetrahydrate. Considering explicit solvent models, relative solvation free energies were calculated by means of the free energy perturbation method through Monte Carlo simulations. These calculated values differ remarkably from those by the PCM approach in aqueous solution, nonetheless the same prevalent conformation was predicted by the two methods. Aqueous solution structure-characteristics were determined by Monte Carlo. Equilibration of conformers/tautomers through water-assisted double proton-relay is discussed. This mechanism is not viable, however, in non-protic solvents where the calculated potential of mean force curve does not predict remarkable solute dimerization and subsequent favorable orientation.

## 1. Introduction

**Scheme 1:**
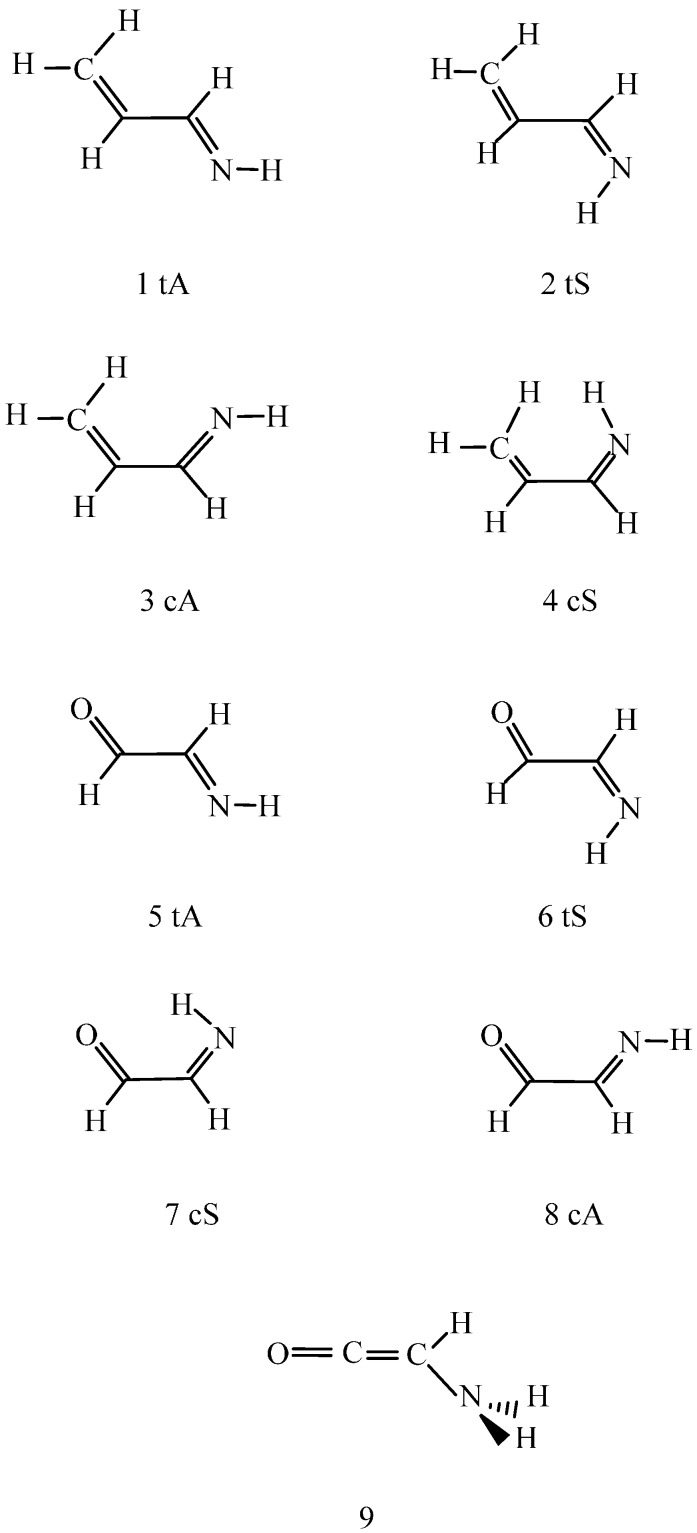
2-Propene-1-imine (**1**–**4**); 2-imino acetaldehyde (**5**–**8**); 2-amino ketene (**9**). Letters t and c stand for the *s-trans* and *s-cis* conformations, respectively, letters A and S stand for CCNH *anti* and *syn* orientations respectively. The heavy atoms are nearly or entirely coplanar. Remarkable deviation from planarity was found for structure (**9**). For detailed geometric data, see Supplementary Information (Table S1 and Figure S1a–d).

**Scheme 2:**
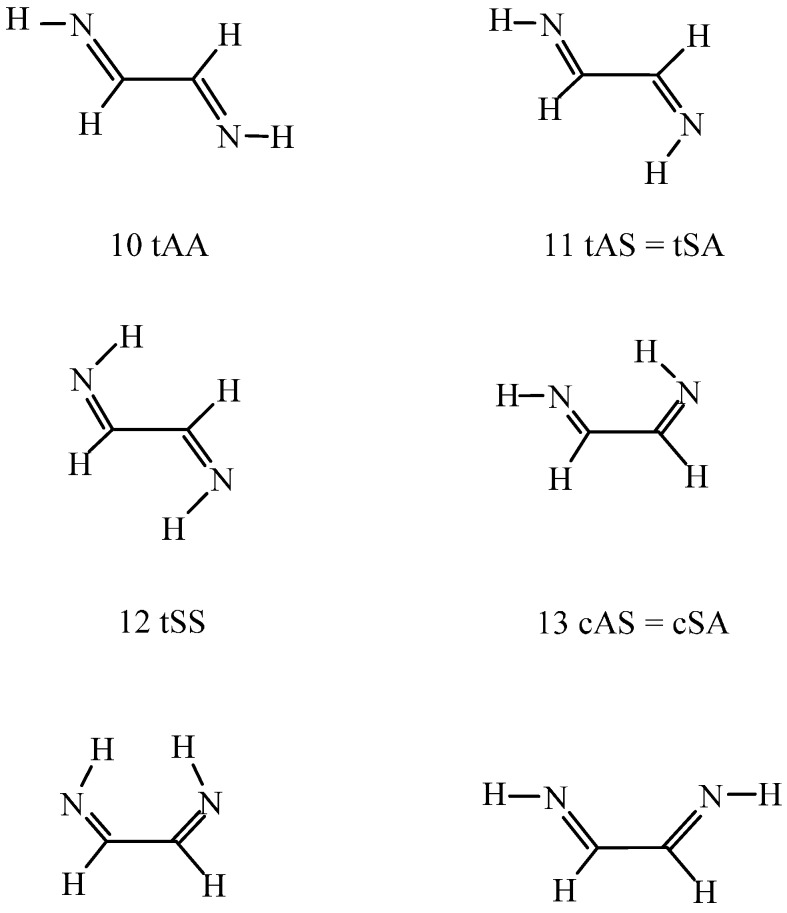
1,2-Ethane diimine (**10**–**15**); 2-amino imino-ketene (**16**); 1,2-diamino acetylene (**17**). For t, c, A and S codes, see Scheme 1. The heavy atoms are nearly or entirely coplanar. Remarkable deviations from planarity was found for structures (**16**) and (**17**). For detailed geometric data, see Supplementary Information (Table S1 and Figure S1a–d).

In a recent publication [1], the conformations of a chain with conjugated double bonds, called double bond-single bond-double bond (DSD) systems, were studied in the gas phase and in different solvents. The central moiety of these structures is the X=C–C=Y fragment, and different combinations of the CH_2_ and O groups for X and Y were investigated previously. The CH_2_, O and NH groups are, however, isoelectronic and can replace each other as X and Y. The scientific aim of the present paper is the structural analysis when one or two NH groups appear in a DSD molecule. For such systems, an *s-trans*/*s-cis* conformational equilibrium is possible, corresponding to XCCY = 180° and 0°, respectively, in combination with the CC=NH *anti* and *syn* arrangements. All structures (Scheme 1 and Scheme 2) have been investigated accordingly.

The R_1_–CH=N–R_2_ structure is the typical subunit in Schiff-bases. If R_1_ contains a double bond in possible conjugation with the indicated C=N double bond, *s-cis*/*s-trans* conformational equilibrium may be expected. This structural peculiarity was demonstrated by Houjou *et al.* for the Schiff bases of double-headed, fused salicylaldehydes [2]. For preparing unsaturated Schiff bases, Sammour *et al.* [3] found that the C=C–C=N substructure is favorable for Michael-type condensation for ring closure, which requires the CCCN *s-cis* conformation. The same substructure appears in an α,β-unsaturated Schiff base, 1-(CH=N–R) cyclohexene, where the hydrolysis mechanism and reaction rate may depend on the conformation of the C=C–C=N moiety [4].

Complexes of Ni and Pd with α-diimine derivatives, including the N=C–C=N substructure have turned out to be important late-metal catalysts for ethylene homo- and co-polymerization [5]. Prerequisite to the complex formation is the *s-cis* conformation of the α-diimine. Conformational analysis for such DSD molecules in solution can be found only rarely in the literature. Exner and Kliegman [6], and references there studied the in-solution conformation equilibrium for RN=CH–CH=NR molecules based on bond moments. The predicted bond moments are questionable, however, in the absence of precise molecular geometries and for the present species with interacting electrons of two π-bonds separated by a formal single bond. Furthermore, bulky R substituents may affect the equilibrium composition. For example, substituted *N*,*N'*-diphenyl α-diimines were studied in [6], where the two benzene rings could favorably interact in the *s-cis* conformation, resulting in additional stability of the conformer not present for the unsubstituted diimine. Thus a high-level quantum chemical study based on in-solution optimized geometries is justified for a possibly better interpretation of the experimental findings. Considering [1] as well, the obtained results are novel on the field of the in-solution conformational analysis for DSD molecules with any combination of the CH_2_, O and NH groups.

In the case when X and/or Y correspond(s) to NH, the conformational variability can be combined with tautomeric isomerization through ketene amine/aldimine and imino-ketene amine/diimine transformations. No theoretical study of the related structural problems, considering also the solvent effects for identifying the most stable conformer/tautomer for a given composition, has been found in the literature either.

Scheme 1 and Scheme 2 show isomeric C_3_H_5_N, C_2_H_3_NO and C_2_H_4_N_2_ structures. Following the ChemSpider (Available online: http://www.chemspider.com) free online chemical structure database, structures (1), (5), and (10) were identified. In general, this database provides experimental and theoretically calculated values of properties for molecules. For species (1), (5), and (10), however, no experimental in-solution data were available, their partition coefficients, basicities, *etc.* were only calculated by means of physical- chemical parameter-predicting softwares.

Thus stable, in-solution species are probably not known experimentally for these simple DSD structures, although the interest toward the prediction of their physical-chemical properties strongly suggests that the molecules are on the horizon of drug-design companies. A structurally related problem emerges for the drugs Nexium and Protonix, where the O=S–C=N substructure appears. The *s-cis*/*s-trans* equilibrium of the two double bonds may influence their biological effect [7].

Since a double-bonded sp^2^ nitrogen bears an in-plane lone pair that can isomerize with the single-bonded group connecting to the nitrogen, the structural variability is even larger. The *syn* and *anti* conformations of oximes (R_1_C(R_2_)=N–OH could isomerize by nitrogen inversion (the lone pair and the N–O bond formally switch), although the reaction path was found to demand activation energy of as large as about 250 kJ/mol in the gas-phase on the basis of 6-31G calculations [8]. However, isomerization activation energy in solution may be much smaller upon some catalytic effect of the solvent having both hydrogen-bond donor and acceptor sites [9,10]. The role of the catalytic water through the keto-iminol tautomerization of related hydroxamic acids was clearly demonstrated by Guruge and Dissanayake [11].

Recently Allen and Tidwell [12] presented a review with a large number of chemical reactions for the formation of substituted ketenes and imino-ketenes. Although structures (9) and (16) were not found among them, the reactions may be successfully applied for their formations, too. 1,2-Diamino acetylene, H_2_N–C≡C–NH_2_ (17) can tautomerize first to a cumulene structure of NH=C=CH–NH_2_ (16), and can move on, in a further step, to the DSD form of HN=CH–CH=NH (10).

Tautomeric transformations through intramolecular pathways are unlikely for the present target molecules for steric reasons. If tautomerization appears in the gas phase at all, the process can take place probably along a complicated path by means of intermolecular interactions. In-solution structural studies for small molecules have been the subject of theoretical investigations for a long time, e.g., [13,14,15,16,17,18,19,20,21,22,23,24,25]. The present paper fits in the series of studying 1,2 disubstituted ethanes [1,16,18,22,23,24] in solution. For substructures with possibly interacting, nearby hydrogen-bonding sites, special molecular mechanics parameterization is needed in order to satisfy demands emerging, for example, through theoretical drug design. The main goal of the present paper is to determine the free energy differences for the structural isomers (conformers/tautomers) of N-containing DSD molecules in solution and providing a clue about the involvement of the solvent in the equilibration process. To this latter aim, supermolecules were investigated in some cases when the continuum solvent approach was applied, and explicit solvent Monte Carlo simulations were performed.

An investigation for a small system allows for the application of high-level theoretical calculations, thus facilitates the exploration of the effects of the applied theoretical level on the results. Accordingly, large-basis-set DFT/B97D [26] relative free energies were calculated for all investigated pairs of conformers/tautomers in the present paper, and the results were compared with the CCSD(T) relative internal energies (coupled-cluster singles and doubles with non-iterative triples [27,28]) at its complete basis set limit in many cases. Former studies [1,23] proved that the IEF-PCM relative solvation free energies, calculated for energy-minimized structures, could remarkably differ from those calculated by the free energy perturbation method utilized in Monte Carlo (FEP/MC) simulations. For comparison, FEP/MC values were also calculated here for several isomeric pairs in dichloromethane and/or in water. This latter type of calculations provides means to characterize the first solvation sphere of the solutes and help explore the thermally averaged possible solute-solvent hydrogen-bond structure.

## 2. Results and Discussions

### 2.1. Gas Phase

Gas-phase energy results are provided in Table 1. Although some of the structures in Scheme 1 and Scheme 2 are rather peculiar, all of them have turned out to form local-minimum-energy tautomers/conformers on the basis of quantum chemical calculations.

The *s-trans*/*anti* form for 2-propene-1-imine (1) is more stable in free energy, ΔE^g^_int_ + ΔG^g^_th_ (see Section 3), by 11.0 kJ/mol than the *s-cis*/*anti* (3) conformation, as calculated at the B97D level. Penn [29] recorded the gas-phase microwave spectrum for this molecule and found only the *s-trans* conformer. An *s-trans* to *s-cis* conformational change requires the rotation about the central C–C bond. The torsion barrier for (1) to (3) is 29–36 kJ/mol at the two theoretical levels (Table 1) with very similar transition state CCCN torsion angles of 95.4° and 96.1°. Although the molecules could acquire the calculated activation energy upon collision for a gas-phase transformation, the experimental result for the lack of the *s-cis* conformer is in good accord with the B97D relative free energy or the calculated CCSD(T)_CBS_ activation energy of 11.4 kJ/mol (the B97D and MP2 ΔG^g^_th_ values could be taken as similar, as revealed from the specifically calculated MP2 values for species (11), see below, thus an estimate for the CCSD(T)_CBS_ relative free energy is 9 kJ/mol, still too high).

**Table 1:** Relative energy/free energy components for tautomeric/conformational isomers ^a^.

Structures in Schemes	Gas		Dichloromethane			Water		
	ΔE^g^_int_	ΔG^g^_th_	ΔE^s^_int_	ΔG^s^_th_	ΔG_solv_	ΔE^s^_int_	ΔG^s^_th_	ΔG_solv_
CH_2_=CH–CH=NH (**1**)	0.0	0.0	0.0	0.0	0.0	0.0	0.0	0.0
TS (**1** to **2**)	113.9	−9.9 ^b^	111.5	−10.1 ^b^	7.8	110.3	−10.3 ^b^	10.3
	123.2 ^c^							
CH_2_=CH–CH=NH (**2**)	2.7	−0.2	2.9	−0.5	−1.8	2.9	−0.5	−2.0
	3.1 ^c^		3.8 ^c^		−2.1 ^d^	3.7 ^c^		−2.3 ^d^
TS (**1** to **3**)								
(95.4°, 96.0°, 96.3°) ^e^	35.5	−3.5 ^b^	35.4	−3.0 ^b^	−0.6	35.5	−3.0 ^b^	−0.9
(96.1°)	29.3 ^c^							
CH_2_=CH–CH=NH (**3**)	13.4	−2.4	14.3	−4.0 ^b,f^	1.3	13.0	−4.0 ^b,f^	2.2
	11.4 ^c^		10.1 ^c^		2.6 ^d^	9.7 ^c^		3.2 ^d^
CH_2_=CH–CH=NH (**4**)	15.1	−2.8 ^b^	16.1	−2.3 ^b^	−8.7	15.1	−2.5 ^b^	−1.4
						13.1 ^c^		−0.9 ^d^
O=CH–CH=NH (**5**)	0.0	0.0	0.0	0.0	0.0	0.0	0.0	0.0
O=CH–CH=NH (**6**)	2.9	−0.5	3.5	0.2	−1.3	3.8	0.0	−2.0
TS (**6** to **7**)								
(87.3°, 88.2°, 88.4°) ^e^	29.1	−3.4 ^b^	29.9	−3.0 ^b^	−3.3	30.4	−3.0 ^b^	−4.5
(89.2°)	30.8							
O=CH–CH=NH (**7**)	7.8	−0.1	8.0	1.0	0.4	8.2	0.4	0.2
	7.8 ^c^		9.6 ^c^		−1.9 ^d^	10.5 ^c^		−3.0 ^d^
O=CH–CH=NH(**8**)	23.1	−5.4 ^d^	26.8	−3.6 ^b^	−10.6	29.0	−3.3 ^b^	−14.9
	24.4		32.5 ^c^		−16.2 ^d^	36.1 ^c^		−22.3 ^d^
O=C=CH–NH_2_ (**9**)	20.8	−1.0	19.7		7.4	19.4	−0.8 ^b^	8.2
	35.1		32.0 ^c^		9.2 ^d^	31.4 ^c^		10.3 ^d^
HN=CH–CH=NH (**10**)	0.0	0.0	0.0	0.0	0.0	0.0	0.0	0.0
HN=CH–CH=NH (**11**)	4.6	−2.3 ^b^	5.8	−2.9 ^b^	−3.4	6.5	−3.0 ^b^	−4.8
	6.2 ^c^		8.7 ^c^	−2.7 ^b^	−5.0 ^d^	9.8 ^c^	−2.9 ^b^	−6.8 ^d^
TS (**11** to **13**)								
(92.7^o^, 93.2^o^, 93.4°) ^e^	32.6	−3.6 ^b^	34.3	−2.8 ^b^	−5.2	35.2	−3.0 ^b^	−7.2
(93.6°)	31.3							
HN=CH–CH=NH (**13**)	11.8	−2.8 ^b^	13.0	−3.3 ^b^	−1.5	13.6	−4.1 ^b^	−2.3
	10.6 ^c^		13.9 ^c^		−4.5 ^d^	15.3 ^c^		−6.2 ^d^
HN=CH–CH=NH (**12**)	6.9	−1.1	7.8	−1.4	−3.6	8.2	−1.7	−4.8
	9.4 ^c^		11.6 ^c^		−4.9 ^d^	12.6 ^c^		−7.1 ^d^
HN=CH–CH=NH (**14**)	16.1		16.7	−0.9 ^b^	−3.0	17.0	−2.2 ^b^	−4.0
			20.8 ^c^		−5.7 ^d^	21.7 ^c^		−7.2 ^d^
TS (**10** to **15**)								
(86.3°, -, 93.5°) ^e^	35.2	−4.0 ^b^				38.3	−3.8 ^b^	−9.8
(83.6^o^)	32.3							
HN=CH–CH=NH (**15**)	28.8		33.3	−4.7 ^b^	−12.8	36.2	−5.4 ^b^	−18.5
			36.7 ^c^		−17.4 ^c^	40.8 ^c^		−24.4 ^d^
HN=C=CH–NH_2_ (**16**)	49.2		49.8		0.1	50.2	−7.5 ^g^	−0.8
	64.0 ^c^							
H_2_N–C≡C–NH_2_ (**17**)	98.6	−7.4 ^g^	99.2		−2.6	99.2	−5.0 ^g^	−4.0

^a^ Values in kJ/mol. For structure numbers in parentheses, see Scheme 1 and Scheme 2. Geometries were optimized at the B97D/aug-cc-pvtz and MP2/aug-cc-pvtz levels in the indicated environment. ΔE_int_ and ΔG_solv_ values (upper rows) from B97D/aug-cc-pvqz single point calculations; ^b^ –*RT* ln2 = −1.7 kJ/mol is included in ΔG_th_ for the entropy of mixing for the TS antipodes or for a symmetry number of 2 for the reference structure; ^c^ CCSD(T)_CBS_//MP2/aug-cc-pvtz energies; ^d^ MP2/aug-cc-pvtz value; ^e^ Values in parentheses for a TS (transition state) stand for the X=C–C=N torsion angles increasing in the gas, CH_2_Cl_2_, and water series (X = CH_2_, O, NH). The energy/free energy parameters are provided with respect to the corresponding data of the CH_2_CHCHNH (1), OCHCHNH (5) and NHCHCHNH (10) conformers, respectively; ^f^ A small imaginary frequency for the lowest energy out-of-plane torsion remained through the energy minimization even using the analytical second derivative optimization. ΔG^s^_th_ was estimated by using the corresponding water frequency (65 cm^−1^), for which the normal coordinate was extremely similar. For all other water vibrations, the frequencies and the normal coordinates were very similar and deviated by 2–4 cm^−1^ for the low-frequency vibrations. The largest deviation has been found at 6 cm^−1^ above 2000 cm^−1^; and ^g^ −2*RT* ln2 = −3.4 kJ/mol is included in the ΔG_th_ because of the rotational symmetry number of 2 for the reference tAA (9) form with C_2h_ symmetry and due to the entropy of mixing for antipodes. TS: transition state.

Penn found, however, two *s-trans* conformers with C–C=N–H *anti* (1) and *syn* (2) arrangements. The latter structure was predicted to be higher in energy by 3.8 ± 0.4 kJ/mol. The calculated relative energy is 2.7 and 3.1 kJ/mol at the DFT and the CCSD(T)_CBS_ levels, respectively. But how can the *anti* and *syn* forms equilibrate in the gas phase?

The experiment was taken at T = 673 K, where a stably existing dimer is unlikely. Furthermore, as will be discussed below, a doubly hydrogen-bonded dimeric structure, preferable for a double proton-relay, is not favored even in dichloromethane solution, where a weak association trend has been still predicted. Then possible intramolecular routes for forming the *s-trans*/*syn* species are H rotation about the C=N double bond or the nitrogen inversion.

B97D transition state geometry optimization for the *s-trans*/*anti* to *s-trans*/*syn* CH_2_CHCHNH, starting from a C–C=N–H torsion angle of 90° and C=N–H bond angle of about 110° as a guess for the potential energy maximum for the H rotation about the C=N bond, led quickly to a TS geometry with C=N–H bond angle of 179.3° and CCNH torsion angle of 124.9°. The torsion angle for the *s*-*trans* CCCN moiety was maintained at 180°. The TS structure was reoptimized at the MP level resulting in CNH angle of 179.2° and CCNH and CCCN torsion angles of 124.5° and 180.0°, respectively (Supplementary Table S1). These results correspond to a combined mechanism for the *s-trans*/*anti* (1) to *s-trans*/*syn* (2) transformation along H rotation about the C=N bond and an increase in the CNH bond angle to almost linear, corresponding to N-inversion. At CNH >179° it is not important what the CCNH torsion angle is. B97D and MP2 predictions of the TS geometry through aug-cc-pvtz optimization are in agreement. The calculated energy barrier is, however, very high, 113.8 kJ/mol and the activation free energy is still about 105 kJ/mol at the B97D/aug-cc-pvqz level. The energy barrier is 123.2 kJ/mol at the CCSD(T)_CBS_ level (Table 1).The average kinetic energy for a gas molecule is 1.5 *RT*/mol = 8.4 kJ/mol at T = 673 K, thus the necessary additional kinetic energy to distort the *s-trans*/*anti* structure to reach the transition state is at least 105.4 kJ/mol. From a Boltzmann distribution, the ratio of the molecules with the necessary additional energy and the average-energy molecules is about 7 × 10^−9^. This is a small ratio, indeed, but even assuming only 1% for the average-energy particles, the number of the molecules with the required activation energy is 6 × 10^21^ × 7 × 10^−9^ ≈ 4 × 10^13^/mol. Thus the fraction is small but the number itself is still large, which, on the basis of the experimental results, could trigger the *anti*/*syn* equilibration.

For the C_2_H_3_NO molecules, the two *s-trans* 2-imino-acetaldehyde structures with *anti* and *syn* positions for the imino hydrogen (5,6) differ by 2.9 kJ/mol in internal energy. The B97D calculations predict the existence of both conformers in the gas phase, although the required activation energy for the equilibration is supposed to be similar to that for the corresponding CH_2_=CH–CH=NH conformers. The relative energies of the *s-cis* conformers (7) and mainly that of (8) are considerable at both theoretical levels predicting nearly equal values. The relative energy of (7) is, however, lower by 3.6–5.6 kJ/mol than that for the structurally related (3) species. How can it be rationalized?

Scheme 1 shows that species (3) cannot have a strong intramolecular hydrogen bond, and could be subject only to a favorable C–H…N interaction with H…N distance of 267 pm. In a recent review [30], problems related to intramolecular *vs* intermolecular hydrogen bonds (mainly in solution for the latter) were surveyed. Upon the 2011 IUPAC (International Union of Pure and Applied Chemistry) recommendations, no clear-cut upper limit was defined for the atom separation in a X…H hydrogen bond, where X is generally an electronegative element. The former “golden rule” was that the X…H bond length must be shorter than the sum of the van der Waals radii of the two atoms [31]. Then the above interaction could be qualified as a hydrogen bond since 267 pm is within this range. An important, but not mandatory feature of a H-bond by IUPAC is the existence of a (3, −1) bond criteria point (BCP). Furthermore, an important feature of a *red-shifting* (classical) X–H…Y hydrogen bond is that the X–H bond length elongates with respect to the reference system without the H-bond and charge is transferred from Y to the X–H. The C–H distance from B97D in (1) is 109.0 pm, the molecular electrostatic potential fitted CHELPG [32] C, H, N charges are −0.337, 0.138, and −0.682 atomic units, respectively. The C–H distance in (3) becomes shorter to 108.8 pm, and the corresponding atomic charges are −0.149, 0.109, and −0.638. Thus quite a remarkable charge was transferred from the nitrogen to the C–H proton. The important finding was that each of the symmetrical and asymmetrical C–H stretching frequencies increased by 10 cm^−1^. Taking the structural information together, the system meets all requirements for a *blue-shifting* hydrogen bonding, which could be, however, weak [33]. From MP2 geometry optimizations, the C–H distance decreases from 108.3 to 108.2 pm and the above net atomic charges are −0.375, 0.155, −0.695 *vs.* −0.189, 0.123, and −0.647. The differences in the sets by the two methods are close.

For the pair of (6) and (7), the B97D/aug-cc-pvtz calculated N–H bond length increases from 102.5 in (6) to 103.1 in (7), the stretching frequency decreases from 3370 to 3296 cm^−1^, and the calculated atomic charges for O, H, N are −0.444, 0.358, and −0656, respectively, in (6) compared with −0.408, 0.317, and −0.562 in (7). The O…H separation is 245.0 pm in (7), the O…H–N bond angle is 105.4°. It is questionable whether such a bent bond still can be assigned to a hydrogen bond category, but the effect of the interactions to the important structural parameters for a red-shifting hydrogen bond formation has been clearly demonstrated. Even if the interaction is only pure electrostatic, the decrease of the relative energy from 11.4-13.4 kJ/mol for (3) to 7.8 for (7) is reasonable.

The transition state activation energy from (6) to (7) was calculated at 29.1–30.8 kJ/mol with OCCN torsion angles of 87.3°–89.2°. The barrier can be overridden upon collision in the gas-phase, and species (7) can be present with (5):(7) ratio of about 4:96 at room temperature. The large relative energy for (8) can be attributed to the repulsion of the nitrogen and oxygen lone pairs existing in the almost planar heavy atom arrangement with OCCN torsion angle of 0.4° (Table S1).

The amino ketene tautomer (9) is higher in energy then species (5) by 20.8 kJ/mol and 35.1 kJ/mol at the DFT and the *ab initio* level, respectively. The calculated values suggest that the predominant conformer/tautomer in the gas phase is structure (5). If the large, possibly about 100 kJ/mol, activation energy can be provided in the gas phase for the formation of (6) (in analogy to the formation of (2), as was proven by the experiment of Penn [29], the calculated (5):(6) equilibrium ratio is 28:72 (the final equilibrium composition depends on all considered species existing in the mixture but the ratios for selected pairs will not change).

For the studied C_2_H_4_N_2_ systems (the isomeric H_2_N–CH_2_–CN amino-acetonitrile was not investigated), the prevalent 1,2-ethanediimine species is the (10, tAA) (see Scheme 2) in the gas phase. It is more stable in free energy by 2.3–5.8 kJ/mol than (11, tAS) and (12, tSS) at the DFT level and must be more stable by a further 2–3 kJ/mol than species (11) and (12) as calculated *ab initio*. The relative energies of the *s-cis* conformers (13–15) were calculated at 11–29 kJ/mol. Relative energies are even much higher for the amino imino-ketene (16) with a cumulated double-bonded structure and the 1,2-diamino acetylene (17). The values calculated at the DFT level are 49 and 99 kJ/mol, respectively. The CCSD(T)_CBS_ relative energy for structure (16) is 64 kJ/mol. The analysis of the energy components in Equation (2) (section 3) revealed that the ΔE^MP2^_CBS_ term is about 90% responsible for the indicated relative energy, whereas (ΔE^CCSD(T)^ − ΔE^MP2^)_aug-cc-pvdz_ accounts for the about 10% remaining. This means that the post-MP2 correction is meaningful for tautomeric systems (5.33 kJ/mol in the present case). The relative post-MP2 correction accounted for an even larger share of the total relative internal energy for species (9). Its contribution to the final value of 35.1 kJ/mol was 10.1 kJ/mol. In contrast, the (ΔE^CCSD(T)^ − ΔE^MP2^)_aug-cc-pvdz_ contributions to the relative conformational internal energies have been found to be small, amounting only to 1–2 kJ/mol in general.

The (11) to (13) conformational change is possible along a rotation about the NCCN bond. The transition state torsion angle was predicted at 92.7°–93.6° by the two theoretical approaches with activation energy of 31–33 kJ/mol. The activation free energy must be smaller by 11% from B97D estimation. When (13) is formed the question can be raised whether there is an N–H…N hydrogen bond. Present computational results cannot give a unique answer. The right-hand side N–H bond length of 102.94 pm in 13 cAS (Scheme 2) shows a very little increase from 102.88 pm in 11 tAS. Scheme 2 clarifies that this N–H bond (CCNH *syn*) can act as the hydrogen bond donor, whereas the left-hand side nitrogen (CCNH *anti*) would be the acceptor atom. The small increase of the *syn* N–H bond in combination with a remarkable charge transfer from the nitrogen in the CCNH *anti* moiety, (atomic charges on this nitrogen are −0.670 and −0.637 in (11) and (13) respectively) suggest a red-shifting hydrogen bond. However, the vibrational frequency for the *syn* N–H bond increases from 3306 cm^−1^ in (11) to 3312 cm^−1^ in (13). Since the bond length increases (even though very slightly), a small decrease instead of the noted small increase in the vibrational frequency would have been expected. For the other N–H bond in the CCNH *anti* substructure, the bond length decreased from 102.37 pm in (11) to 102.21 pm in (13) and the concomitant frequency change was an increase from 3377 to 3398 cm^−1^. The N…H distance was calculated at 245.9 pm at the B97D level with N–H…N bond angle of 105.6°. These geometric data would still comply with a strongly bent regular H-bond, although it was unable to identify the hydrogen bond character in the gas phase on the basis of the above contradictory data. In contrast, the stretching frequency of the donor N–H in the *s-cis*/*syn* conformation decreased by 10 and 20 cm^−1^ in dichloromethane and water, respectively, completing the required conditions for a red-shifting intramolecular hydrogen bond.

Finally, the transformation of species (10) to (15) was studied through rotation about the C–C bond. The NCCN torsion angle is 83.6°–86.3° in the transition state with energy 32–35 kJ/mol above that for the reference (10) conformation. The two theoretical methods thus provide similar results. Conformer (15) corresponds to a NCCN *s-cis* structure with two HNCC *anti* moieties (cAA in Scheme 2). Although the molecule is not planar, the NCCN torsion angle of 26.8° could only partially diminish the repulsion of the nitrogen lone pairs. Consequently, the relative energy is high, 28.8 kJ/mol at the B97D level and the conformer is not expected to appear in the gas-phase equilibrium mixture.

Whereas no comparison of the calculated relative energies for the studied C_2_H_4_N_2_ systems is possible in the absence of experimental data for 1,2-ethanediimine itself, Hargittai and Seip [34] investigated the molecular structure of its *N*,*N'*-*di*-tertiary butyl derivative by gas electron diffraction. The predominant structure was found *gauche* with N=C–C=N torsion angle of 65° by a rotation from the *syn* form (corresponding to *s-cis* in this paper), although a small fraction of the *anti* (*s*-*trans*) conformer was also assigned. The present author attributes this experimental result mainly to the favorable dispersion interactions of the two bulky t-butyl groups in the *gauche* rather than in the *s-trans* form, and the decrease of the relative free energy by *RT* ln2 = 2.03 kJ/mol at the temperature of the experiment (~353 K) due to the mixing of the two optical antipodes emerging in the case of a *gauche* structure.

A structure, closely related to 1,2-diamino acetylene, μ_2_,η^2^-1,2-diaminoethylene was characterized by using Fourier transform-reflection absorption infrared spectroscopy (FT-RAIRS) as a surface species [35]. The C atoms in this structure are bound to a Pt surface and the spectra predicted a H_2_N–C–C–NH_2_ moiety, with delocalized π electrons along the NCCN path. Two different amino N–H stretching frequencies were assigned according to the C_2v_ symmetry. The relevance of this result with respect to the present study is that no imine frequencies were assigned. Thus the H_2_N–C≡C–NH_2_ tautomer could be also stable in the gas phase in spite of its about 99 kJ/mol relative energy.

### 2.2. In-Solution Results

Table 1 summarizes the results of the quantum mechanical calculations for solutes in dichloromethane and water solvents, as well. Internal energy differences and IEF-PCM/ΔG_solv_ values were obtained from B97D/aug-cc-pvqz//B97D/aug-cc-pvtz single point calculations in every case. In a number of cases, relative internal energies were calculated from CCSD(T)_CBS_ values following the IEF-PCM/MP2/aug-cc-pvtz geometry optimization. The related ΔG_solv_ values were obtained at this optimization level.

The energy of a structure, E^g^_int_, is lowest at a theoretical level if the geometry is optimized in the gas phase. If the molecule is imbedded in a solvent environment, its internal energy will increase: E^s^_int_ becomes less negative than E^g^_int_ in order to make G_solv_/PCM optimally negative. The SCF (self-consistent field) procedure through the IEF-PCM in-solution geometry optimization stops where the (E^s^_int_ + G_solv_) term reaches a local minimum. Increase of the internal energy has two sources: the geometry distortion as compared to the gas-phase structure and the solute polarization. Table S1 shows that the main geometric parameters change only slightly due to solvation, thus the increase of E_int_ in solution must be attributed mainly to polarization effects. In a former study, Alagona *et al.* [36] calculated the separate effects of the two contributions for small molecules, and the polarization effect was found to be of significantly larger importance in DFT calculations.

ΔE^s^_int_ from IEF-PCM/B97D calculations are both larger and smaller than the corresponding gas-phase values by 1–2 kJ/mol in general. It is worth mentioning that this feature of the *relative* internal energy is allowed theoretically upon solvation, whereas the individual E_int_ values always increase. The variation of the in-solution ΔE^s^_int_ value in comparison with its gas-phase counterpart, ΔE^g^_int_, depends on which species undergoes smaller energy increase upon solvation. The smaller increase in E_int_ for the more stable form leads to an increased ΔE^s^_int_ and *vice versa*.

Remarkable increases in ΔE_int_ were obtained for structures (8) and (15) with outstandingly large solvent effects in water. Since ΔG_solv_/PCM is conspicuously negative, the concomitant ΔE^s^_int_ values increased by 5.9 and 7.4 kJ/mol as compared with ΔE^g^_int_ of 23.1 and 28.8 kJ/mol, respectively, suggesting considerable internal energy increases for structures (8) and (15) in aqueous solution.

Bond lengths and bond angles change negligibly upon solvation. In contrast, the XCCY torsion angles for heavy atoms significantly deviate from 0° or 180° in solution for species (4), (8), and (15). The heavy atoms in tautomers (9) and (16) are far from coplanarity even in the gas phase. The solvation changes the XCCY torsion angles only slightly. Indicated HNNH torsion angles for (17) in Supplementary Table S1 also do not change remarkably leaving the structure without any symmetry.

The in-solution ΔG^s^_th_ values are generally moderate in comparison with the corresponding ΔE^s^_int_ values. The gas-phase molecular symmetries were preserved in solution, thus the related entropy contributions to the corresponding G^s^_th_ values still hold. The sign of the ΔE^s^_int_ + ΔG^s^_th_ + ΔG_solv_/PCM sum was always dominated by the sign of the ΔE^s^_int_ term. Accordingly, the sign of the total relative free energy, ΔG^s^_tot_ is equal to that for ΔE^s^_int_ in every case.

IEF-PCM/CCSD(T)_CBS_ calculations predicted that ΔE^s^_int_ would increase for the conformers by up to 7.1 kJ/mol (except (3) and (4) with a decrease up to 4.2 kJ/mol) as compared with the B97D/aug-cc-pvqz result for structures in dichloromethane and water, and the increase by about 12.0 kJ/mol for species (9) is similar to that found for this latter tautomer in the gas-phase. The most surprising result was, however, that the IEF-PCM/MP2/aug-cc-pvtz ΔG_solv_ for (7) was significantly negative in comparison with the small positive value by the DFT calculations. In other cases, the MP2/aug-cc-pvtz relative solvation free energies preserved at least the sign of ΔG_solv_/PCM as calculated by B97D. In general, if the *ab initio* ΔE^s^_int_ increases, the related MP2/ΔG_solv_ becomes more negative for the conformers and *vice versa.* The deviations call attention to the probably different account for the solute polarization by the two theoretical methods. For tautomer (9), however, both ΔE^s^_int_ and ΔG_solv_/PCM increase.

IEF-PCM relative solvation free energies for conformational local energy minima scatter in a wide range of 3.2 and –24.4 kJ/mol, whereas those for tautomers (9,16,17) vary between 10.3 and −4.0 kJ/mol. The remarkable cases from the point of view of the estimation of the equilibrium composition are when the ΔE^s^_int_ and ΔG_solv_ values are of opposite signs, thus when ΔG_solv_ is negative in Table 1 (with positive ΔG_solv_/PCM, the species is even less preferable than calculated on the basis of the ΔE^s^_int_ + ΔG^s^_th_ term). For CH_2_=CH–CH=NH (2), ΔG^s^_tot_ from B97D is only 0.4–0.6 kJ/mol, considerably smaller than the gas-phase value of 2.5 kJ/mol. The reduction means that the (2), the *s-trans/syn* fraction is 44%–46% of the total *s-trans* form in solution at room temperature. For O=CH–CH=NH (6), also at T = 298 K, the B97D ΔG^s^_tot_ values are 2.4 and 1.8 kJ/mol in dichloromethane and water, respectively, corresponding to 28:72 and 33:67 molar fractions in the respective solvents for the less and more stable *s-trans* conformers with the H–N bond pointing in different directions. The ratio is about 24:76 in the gas phase as calculated from energies, thus the solvent effects are small for the *s-trans* 2-imino-acetaldehyde.

The most stable conformation of 1,2-ethanediimine seems to be a delicate question at the IEF-PCM level. Whereas (10), the *s-trans*/*anti*/*anti* conformation is clearly the most stable in the gas phase, ΔG^s^_tot_ for the (11) *s-trans*/*anti*/*syn* conformation is −0.5 and −1.3 kJ/mol in dichloromethane and water, respectively, at the B97D level. For a comparable estimation, the time-consuming MP2/aug-cc-pvtz frequencies were calculated for the corresponding pair, showing that the derived ΔG^s^_th_ values hardly deviated from the B97D values in the four cases studied. This finding supports that one may use the B97D values in other cases, as well, when the relative CCSD(T)_CBS_ free energies are to be calculated. For the present case, (10) is just slightly more stable than (11) at the *ab initio* level even in aqueous solution. Nonetheless, the problem will be studied using the FEP/MC method and discussed below.

In the *s-cis/anti*/*anti* conformation of HN=CH–CH=NH (15), when the two lone pairs of the nitrogen atoms point mostly toward each other, the relative internal energy largely increases compared with (13) and (14). Although the considerably more negative ΔΔG_solv_/PCM values in water indicates that the solvation strongly supports the formation of this conformer, ΔG^s^_tot_ remains too positive preventing the observable appearance of this conformer in the equilibrium mixture.

ΔG_solv_/PCM values are negative for the TS structures regarding rotations about the central C–C bond. All calculated XCCN torsion angles for such conformer transformations are about 90°. Apparently, solvation favors the nearly perpendicular rather than coplanar arrangements for the XCC and CCY planes of the heavy atoms. In contrast, ΔG_solv_/PCM for the TS corresponding to the combined H rotation about the C=N bond/N-inversion for the *s-trans* CH_2_=CH–CH=NH is strongly positive, with coplanar CCCN skeleton.

**Figure 1:**
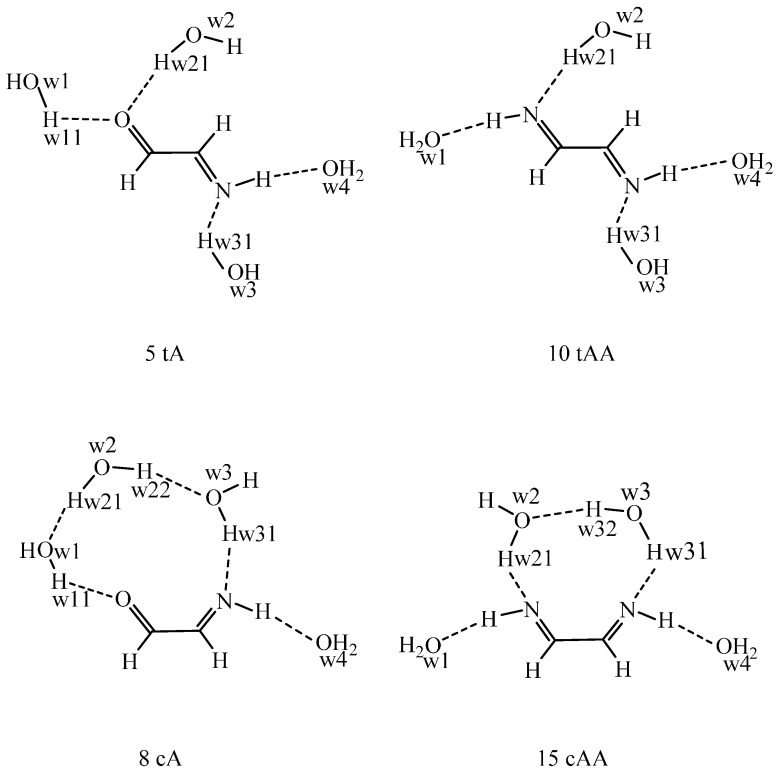
The structures of the tetrahydrates of the *s-trans* species 5 and 10 and the *s-cis* species 8 and 15 as optimized in continuum water solvent at the IEF-PCM/B97D/aug-cc-pvtz level. The hydrogen bond parameters (from left to right) in Figure 1 are as follow. 5 tA: OCCN = 180°, the O=C, O…H_w11_ (198 pm), and O…H_w21_ (201 pm) bonds are coplanar, bond angles: O_w1_H_w11_…O = 177°, O_w2_H_w21_…O = 178°. The C=N, N–H and the N…H_w31_ (187 pm) bonds are coplanar, H…O_w4_ = 208 pm. The N…H_w31_O_w3_ bond angle is 177°, N–H…O_w4_ = 173°. 10 tAA: NCCN = 179.9°, the N=C, N–H, and N…H_w21_ (187 pm) bonds are coplanar, H…O_w1_ = 209 pm. Bond angles: O_w1…_H–N = 173°, O_w2_H_w21_…N = 177°. The C=N, N–H and the N…H_w31_ (187 pm) bonds are coplanar, H…O_w4_ = 208 pm. The N…H_w31_O_w3_ bond angle is 177°, N–H…O_w4_ = 173°. 8 cA: OCCN = 23.7°, only one water—carbonyl hydrogen bond: O…H_w11_ (194 pm), O_w1_H_w11_…O = 161°. Waters 1 and 2 form a solvent-solvent hydrogen bond with O_w1_…H_w21_ distance of 194 pm and O_w1_…H_w21_O_w2_ angle of 177°. Water 2 and water 3 are also linked by a solvent-solvent hydrogen bond: H_w22_…O_w3_ = 187 pm and O_w2_H_w22_…O_w3_ = 177°. The C=N, N–H and the N…H_w31_ (186 pm) bonds are almost coplanar (the sum of the bond angles is 359°), H…O_w4_ = 201 pm. The N…H_w31_O_w3_ bond angle is 178°, N–H…O_w4_ = 176°. 15 cAA: NCCN = 30.5°, the N=C, N–H, and N…H_w21_ (182 pm) bonds are coplanar, H…O_w1_ = 207 pm. Bond angles: O_w1…_H–N = 176°, O_w2_H_w21_…N = 177°. Water 2 and water 3 form a solvent–solvent hydrogen bond: O_w2_…H_w32_ = 193 pm and O_w2_…H_w32_O_w3_ = 161°. The C=N, N–H and the N…H_w31_ (190 pm) bonds are coplanar, H…O_w4_ = 210 pm. The N…H_w31_O_w3_ bond angle is 175°, N–H…O_w4_ = 172°.

A well-known shortcoming of the continuum dielectric solvent model is that it may underestimate the solvation effects on the possible solute-solvent hydrogen bonds, when the pure solute is placed into the cavity carved within the solvent. For improving the performance of the IEF-PCM, a supermolecule may be considered, where the solute is surrounded by a number of water molecules and this supermolecule is embedded into the cavity within the continuum solvent. To this aim, tetrahydrate models have been studied for two solutes in two conformations for each (Figure 1). By an explicit solvent Monte Carlo method, the thermally averaged solute-solvent hydrogen bond interactions can be considered. In the present, classical MC, the solute has to be characterized by preset net atomic charges, which can be derived by a fit to the in-solution molecular electrostatic potential (Table 2).

In the optimized tetrahydrate structures, the OCCN and NCCN torsion angles are about 180° for 5 tA and 10 tAA in accord with their values for the pure solute in the water cavity, whereas the torsion angles for 8 cA and 15 cAA increase by 12°–17° as compared with their B97D values in Table S1. There are four solute-solvent hydrogen bonds to 5 tA, 10 tAA, and 15 cAA. In 8 cA a water-water hydrogen bond (w1…w2) comes into existence instead of a =O…H_w21_O_w2_ bond. The hydrogen bonds are shorter when the water hydrogen is the donor than when a N–H…O_w_ bond is formed.

The problems related to the supermolecule approach when considering a limited number of water molecules becomes evident by the structures in Figure 1. The four hydrogen-bonding sites are far from each other in 5 tA and 10 tAA and the connecting water molecules cannot form a water-water hydrogen bond in parallel with the formation of solute-water hydrogen bonds. In the *s-cis* conformations, a three-member water chain is formed for 8 cA and a water-water hydrogen bond is stable in 15 tAA. As a result, the *s-cis* imino-aldehyde tetrahydrate (8 cA) is more stable than the *s-trans* (5 tA) tetrahydrate by 2.7 kJ/mol, in comparison with the relative ΔE^s^_int_ + ΔG_solv_/PCM energies of 29.1–14.9 = 14.2 kJ/mol for the pure 8 cA solute at the IEF-PCM/B97D/aug-cc-pvtz level (Table 1 contains aug-cc-pvqz values). The tetrahydrate result is a consequence of the two water-water hydrogen bonds along the three-water chain. Their BSSE-corrected interaction energy in the gas phase (no BSSE available in IEF-PCM) is 25.4 kJ/mol. If the relative tetrahydrate energy is corrected by this value, the *s-cis* 8 cA system becomes 22.7 kJ/mol less stable than the 5 tA tetrahydrate vs 14.2 kJ/mol for the pure solutes. This correction is justified, because the water bridges, affecting the total energy only for 8 cA, specifically emerge for this structure due to considering only a limited number for explicit water molecules.

In a real system, each water molecule in the first hydration shell is in hydrogen bond(s) with waters in the second shell or with those around the CH sites, thus the discussed peculiarity will not be relevant. In an aqueous solution, w1, w2, and w3 of 5 tA are also in hydrogen bonds with its neighbors, and w_4_ for both conformers, too. In conclusion, the reversal of the relative energy is the consequence of considering only four water molecules in the supermolecule.

**Table 2:** B97D/aug-cc-pvtz polar atomic and net solute charges from IEF-PCM calculations ^a^.

Structures in Schemes	Polar Atoms	Pure Solute	Solute + 4H_2_O	Net Solute Charges	
				Pure Solute	in Supermolecule
O=CH–CH=NH (**5**)	O	−0.510	−0.340	0.000	0.268
	N	−0.754	−0.495		
	H	0.419	0.406		
O=CH–CH=NH (**8**)	O	−0.488	−0.389	0.000	0.184
	N	−0.726	−0.489		
	H	0.404	0.368		
HN=CH–CH=NH (**10**)	H	0.404	0.460	0.000	0.186
	N	−0.800	−0.666		
	N	−0.800	−0.641		
	H	0.404	0.439		
HN=CH–CH=NH (**15**)	H	0.388	0.354	0.000	0.208
	N	−0.757	−0.531		
	N	−0.757	−0.479		
	H	0.388	0.325		

^a^ For structure numbers in parentheses, see Scheme 1 and Scheme 2. Geometries were optimized at the IEF-PCM/B97D/aug-cc-pvtz level in aqueous solution. Charges were derived by their CHELPG fit the in-solution molecular electrostatic potentials.

The same analysis can be performed for the 1,2-ethanediimine conformers. In this case, 10 tAA is still more stable than 15 cAA by 9.8 kJ/mol in comparison with ΔE^s^_int_ + ΔG_solv_/PCM of 36.3–18.6 = 17.7 kJ/mol for the pure solutes. The correct preference was still maintained because there is only one water-water hydrogen bond in 15 cAA, for which the BSSE corrected interaction energy is –11.0 kJ/mol. Considering this term, 10 tAA becomes more stable than 15 cAA by 20.8 kJ/mol, near the relative in-solution energy for the pure solutes. In the transition state, no water-water hydrogen bond formation was noticed. The NCCN torsion angle was found as 95.1° in comparison with 93.5° for the pure solute. The tetrahydrate transition state is higher in energy by 27 kJ/mol compared with the calculated value of 38.3 − 9.8 = 28.5 kJ/mol for the pure solute. The agreement is very good; consideration of explicit water molecules for the transition state along the *s-trans* to *s*-*cis* transformation results in only a small energy decrease.

As a partial summary, supermolecule models in a continuum dielectric solvent may distort the relative energies if only a limited number of water molecules are considered. In some solute conformations the water molecules may form water-water hydrogen bonds, whereas such bonds do not appear in the case of other solute structures. The water-water hydrogen bond interactions make the related supermolecule energy artificially too negative. This phenomenon typically emerges for conformational equilibrium calculations of small molecules with two, near polar sites open to form solute-water hydrogen bonds. If the relative energies are approximately corrected by the water-water hydrogen-bond interactions existing in one but not in another conformation, the calculated relative energies differ by less than 8 kJ/mol in comparison with the pure solute in-solution energies in the cases studied here at the B97D/aug-cc-pvtz level.

Nonetheless, the optimized tetrahydrate structures can well be compared with the derived first hydration shell structure of different conformers to be discussed below. Those results stem from Monte Carlo simulations, for which IEF-PCM derived net atomic charges have been utilized (Table 2).

In a hydrogen bond, charges are always transferred from the acceptor to the donor [33]. Since the X…H distances are shorter in the X…HOH hydrogen bonds (X = N, O) than in the N–H…O (water) bonds, the former bonds must be stronger and consequently larger amount of charges are to be transferred from the solute to the water molecules than in the opposite direction through the formation of the N–H…O (water) bonds. The total solute charge is zero for a pure solute in the continuum solvent. The total supermolecule charge is also zero but the net solute charge, due to charge transfers, is generally not zero. Depending on the direction of the net charge transfer, the solute can be both positive and negative, As discussed above, more charge are expected to leave the present solutes than being received from the surrounding water molecules, thus the net solute charge should be positive. Numerical results in Table 2 prove it.

Based on the above numerical values, the tetrahydrate solute charges cannot be utilized in atomic charge parameterization for MC because an essential requirement is the total zero charge for an explicit-solvent solution model. Table 2 shows that the net solute charge from the tetrahydrate is strongly positive. Since the atomic charges for the applied TIP4P model are fixed and sum up strictly to zero (although the molecule is polarized in comparison with the gas-phase water for producing good density and heat of vaporization for the liquid water [37]), only the charges derived for the pure solute could be used in the present calculations.

By the FEP/MC procedure [38,39], the relative solvation free energy was calculated for different conformational/tautomeric transformations. The results are compared with the corresponding IEF-PCM/B97D (upper row) and IEF-PCM/MP2 (lower row) values in Table 3. The goal of the presented MC calculations is not to completely repeat the corresponding IEF-PCM solvation free energy calculations. Results in Table 3 were intended to point out that consideration of explicit solvent molecules, primarily water molecules would affect the derived ΔG_solv_ due to supposedly account for the thermally averaged solute-water hydrogen bond interactions in the first hydration shell.

Regarding the B97D results in the table, the ΔG_solv_/PCM values as calculated by using the aug-cc-pvtz basis set are also provided in parentheses. These values deviate from the corresponding aug-cc-pvqz values within the rounding error. Thus the difference of the MP2/aug-cc-pvtz and B97D/aug-cc-pvtz ΔG_solv_/PCM values should be attributed to the applied method, just like for ΔE^s^_int_ as discussed above.

The FEP/MC simulations utilized the IEF-PCM/B97D/aug-cc-pvqz molecular electrostatic potential fitted charges. The calculated ΔG_solv_ values for the *s-trans/anti* to *syn* conformational change (1 to 2) by the IEF-PCM and MC methods are close in aqueous solution, suggesting that the solute is similarly well exposed to hydration, and formation of solute-water hydrogen bonds are favored in both conformations (Table 4). For other conformer pairs, however, the situation is apparently largely different. For (7) *vs.* (5) and (13) *vs.* (12), the possible intramolecular hydrogen bond (see for the discussions above) must strongly reduce the solute’s capacity for forming solute-solvent hydrogen bonds and the MC value is then largely increased. Tautomeric change to structure (9) is not supported in any solvent by any method. The MC result is outstandingly unfavorable in water. In contrast, aqueous solvations of (8) and (15) are highly favored at any level, even though the MC values are remarkably less negative than those from IEF-PCM. The structural basis for the favorable solvation is that the *anti* HNCC arrangement allows favorable hydration of the N–H bonds, whereas the nitrogen lone pairs are also open to accept a hydrogen bond with a nearby water molecule. The O…N and N…N distances are 285 and 290 pm in (8) and (15), respectively. There is room enough for locating 1-2 hydrogen bond donor water molecules. Nonetheless, ΔE^s^_int_ is too high for each of these species and prevents their appearance in an aqueous solution

**Table 3:** IEF-PCM and FEP/Monte Carlo relative solvation free energies, ΔG_solv_^a^.

For Transformation	Dichloromethane		Water	
	IEF-PCM	MC	IEF-PCM	MC
CH_2_=CH–CH=NH				
**1** to **TS** (toward **2**)	7.8 (7.8)	5.1 ± 0.1		
**1** to **2**	−1.8 (−1.8)	−1.2 ± 0.2	−2.0 (−2.1)	−1.6 ± 0.3
	−2.1		−2.3	
O=CH–CH=NH				
**5** to **7**	0.4 (0.4)	1.4 ± 0.5	0.2 (0.3)	9.5 ± 0.9
	−1.9		−3.0	
**5** to **8**			−14.9 (−14.8)	−9.0 ± 0.3
			−22.3	
**5** to **9**	7.4 (7.4)	5.7 ± 0.2	8.2 (8.2)	14.1 ± 0.4
	9.2		10.3	
HN=CH–CH=NH				
**10** to **11**	−3.4 (−3.3)	−0.8 ± 0.3	−4.8 (−4.8)	−0.1 ± 0.4
	−5.0		−6.8	
**10** to **12**			−4.8 (−4.8)	2.6 ± 0.4
			−7.1	
**10** to **TS** (toward **15**)			−9.8 (−9.8)	−3.1 ± 0.2
**10** to **15**			−18.5 (−18.6)	−9.4 ± 0.4
			−24.4	
**12** to **13**	2.1 (2.1)	4.5 ± 0.3	2.5 (2.5)	10.2 ± 0.5
	0.4		0.9	

^a^ Values in kJ/mol. IEF-PCM: upper row B97D/aug-cc-pvqz (aug-cc-pvtz values in parentheses), lower row MP2/aug-cc-pvtz values.

Table 3 shows that the calculated B97D and the average MC relative solvation free energies differ by 0.6–2.7 kJ/mol in dichloromethane and the ΔG_solv_ signs always agree. The difference scatters between 0.4 and 9.3 kJ/mol for aqueous solutions and the signs calculated by the two methods agree in all cases but for ΔG_solv_ (10) to (12). This result, 2.6 ± 0.4 makes the relative free energy of the *s-trans*/*syn*/*syn* conformer much less stable than calculated for the pure solute; ΔG_tot_ of 1.7 kJ/mol from Table 1 increases to 9.1 kJ/mol. The result predicts only a small fraction for the *s-trans*/*syn*/*syn* 1,2-diiminoathane conformer (12) in aqueous solution. The FEP/MC value is −0.1 ± 0.4 for the (10) to (11) transformation and would be −1.3 kJ/mol at the lower limit at the 3SD (99%) level, which is not enough to stabilize (11) relative to (10). ΔG_tot_ of −1.3 kcal/mol from Table 1 increases to 2.2 kJ/mol on the basis of the MC calculations. A relative free energy of 2.2 kJ/mol corresponds to (11): (10) ratio of 29:71 at room temperature.

**Table 4:** Coordination numbers (CN) and number of hydrogen bonds (*n*_HB_) in aqueous solution ^a^.

Structures in Schemes	O/O_w_	O/H_w_	N_t_/O_w_	N_t_/H_w_	N_c_/O_w_	N_c_/H_w_	(N)H_t_/O_w_	(N)H_c_/O_w_	*n*_HB_^b^
CH_2_=CH–CH–NH (**1**)			3.4	2.0			0.9		2.3 (−3.0) ^c^
CH_2_=CH–CH–NH (**2**)			3.1	2.1			0.8		2.4 (−3.0) ^c^
O=CH–CH=NH (**5**)	1.4	1.3	2.5	1.3			1.0		2.9 (−3.0)
O=CH–CH=NH (**7**)	- ^d^	1.1			2.0	1.0		0.8	1.4 (−3.5) ^c^
O=CH–CH=NH (**8**)	1.9	1.5			2.8	1.3		1.0	3.0 (−3.5)
O=C=CH–NH_2_ (**9**)	- ^d^	0.6 ^e^	- ^d^	1.0			0.7		2.3 (−2.5) ^c^
HN=CH–CH=NH (**10**)			2.8	1.5			0.8		3.9 (−3.0)
HN=CH–CH=NH (**11**)			2.9, 3.1	1.3, 1.7			1.0, 1.0		4.4 (−3.0)
HN=CH–CH=NH (**12**)			3.0	1.5			0.95		4.1 (−3.0)
HN=CH–CH=NH (**13**) ^f^					2.1, 2.7	1.3, 1.0		0.4, 0.95	2.6 (−3.0)
TS (from **10** toward **15)**^f^			3.2	1.6				1.0	4.3 (−3.0)
HN=CH–CH=NH (**15**) ^g^					2.9	1.6		1.0	4.7 (−2.5)

^a^ For species 9, the nitrogen/X CN values appear with subscript “t”, for 11 and 13 coordination numbers refer to nitrogens from the left to right in Scheme 2. Integration limits: 305 pm (O/O_w_), 240–255 pm (O/H_w_), 325–355 pm (N/O_w_), 245–260 pm (N/H_w_), 235–255 pm (H/O_w_); ^b^ Integration limit of the pedf in parentheses; ^c^ No resolved first peak below 350 pm; ^d^ Middle of a plateau; ^e^ End of a plateau at 225 pm; ^f^ First and second CN values for the right-hand and the left-hand side N–H atoms, respectively; and ^g^ Equivalent nitrogens in gauche position.

Since in any case but one the signs for ΔG_solv_ have been preserved when the IEF-PCM/B97D and the MC values are compared, consideration of the ΔG_solv_/MC values would not modify the ΔG_tot_ results qualitatively as obtained by IEF-PCM (not even for (12)). In other words, the MC studies do not lead to the reversal of the preferred structure for the studied pairs. For the *s-cis* O=CH-CH=NH conformer (7), even the IEF-PCM study predicted a fraction of only about 3% in the equilibrium composition in aqueous solution (whereas there is about 32% (6) also present as calculated from IEF-PCM values). On the basis of the MC results, the fraction must be further reduced. Each of the relative ΔE^s^_int_ and ΔG_solv_ is so much positive for O=C=CH–NH_2_ (9) in both solvents (Table 1) that this tautomer would not appear in the solution except if the kinetic control is in effect (see next section). For conformer (13), its fraction relative to the most stable (10) *s-trans* form is about 3%–5% in both solvents upon the IEF-PCM calculations. On the basis of the FEP/MC simulations, this fraction practically disappears in solution. Thus each of the two models predicts small, possibly negligible fractions even for the most stable *s-cis* conformations in the studied solutions.

### 2.3. Solution Structures

By utilization of the MC simulation results, some structural characteristics of the considered species in aqueous solution are summarized in Table 4. The coordination numbers (CN) were calculated by integration of the radial distribution functions (rdf) [40] up to their first minima, as indicated in the footnote of the table. The sites of the first minima scatter within a few tens of a pm. The *n*_HB_ values were obtained by integration of the solute-solvent pair-energy distribution functions (pedf) up to their first minima or until the middle of a plateau. According to Jorgensen *et al.* [37], *n*_HB_ may be considered as the number of the solute-solvent intermolecular hydrogen bonds in a protic solvent.

The CN and *n*_HB_ values are very similar for the *anti* (1) and *syn* (2) *s-trans* CH_2_=CH–CH–NH conformers. The slightly larger *n*_HB_ number for the *syn* form and its dipole moment of 3.5 D *vs* 2.9 D for the *anti* form, calculated from point charges, are in accord with the −1.5 kJ/mol for ΔG_solv_ by MC. The B97D exact dipole moments are 3.6 and 3.0 D, respectively. In general, the point-charge-based dipole moments reproduced the exact values (Table S1) generally within 0.2 D for the studied species.

While hydration characteristics of the *s-trans/anti* (5) and *s-cis*/*anti* (8) (for which conformers tetrahydrates were also investigated in Figure 1) are rather similar, coordination numbers for *s-cis/syn* (7) 2-imino-acetaladehyde and for the amino-ketene tautomer (9) are rather different. The first solvation sphere around the carbonyl oxygen is well defined for (5) and (8) with O/O_w_ coordination numbers of 1.4–1.9. Comparing the O/O_w_ and O/H_w_ CN values for (5), the CN’s are nearly equal suggesting that every solvating water molecule forms one hydrogen bond pointing toward the carbonyl oxygen. O/H_w_ is less by 0.4 units than O/O_w_ for (8), suggesting that not all surrounding waters form hydrogen bonds with the carbonyl oxygen. This is in good accord with the 8 cA tetrahydrate structure prediction in Figure 1, where water 2 is bound to water 1 instead of forming a =O…H_w21_O_w2_ hydrogen bond. No resolved O/O_w_ peaks were noted, however, for the other two C_2_H_3_NO species. There is an intramolecular hydrogen bond in the *s-cis* conformer (7) and whereas the nitrogen can act as a full hydrogen-bond acceptor (N_c_/H_w_ = 1.0), the (N)_c_H proton can only partially act as a hydrogen-bond donor to a water oxygen as concluded from (N)_c_H/O_w_ coordination number of 0.8, thus less than 1 for a full hydration. As a consequence, the *n*_HB_ value decreased from 2.9 for (5) to 1.4 for (7). Although the dipole moment is slightly larger for the latter (Supplementary Table S1), the relative solvation free energy is remarkably positive, 9.5 ± 0.9 kJ/mol from MC simulations, probably due to the smaller number of the hydrogen bonds. The O/H_w_ CN for the ketene oxygen (9) is considerably smaller, 0.6 compared with 1.3 for (5), indicating a less strictly localized solvent sphere at this site. In contrast, the N_t_/H_w_ as well as the (N)_t_H/O_w_ coordination numbers for the two tautomers are much closer to each other, suggesting no basic hydration difference for the =NH and the –NH_2_ groups. There are 1.0–1.3 water hydrogens around the nitrogen atoms for (5) and (9), which can form strong N…H_w_O_w_ hydrogen bonds. Overall, the reduced *n*_HB_ number relative to that for (5) and the remarkably smaller dipole moment for (9) support the calculated ΔG_solv_ of 14.1 ± 0.4 kJ/mol.

The HN=CH–CH=NH *s-trans* (10) and (12) conformers have a C_2h_ symmetry, whereas conformers (11) and (13) have only a C_s_ symmetry. The CN values (subscript “t” for (10–12) and “c” for (13)) must be very close for the equivalent nitrogens and hydrogens in structure (10) if statistical fluctuation is accepted (only the average of the almost equal individual values are presented). The same expectations apply for (12). The N–H groups are not identical in (13), for this species two values are presented. For conformers (10–12), the nitrogen atoms are open to hydration by water at their lone pair regions, and the imine hydrogens are also easily reachable by water oxygens. All these hydration patterns can assure formations of N…H_w_O_w_ and N–H…O_w_ intermolecular hydrogen bonds. For conformer (13), the N_c_/O_w_ and the (N)_c_H/O_w_ values differ considerably from the corresponding N_t_ CNs. The differences stem from the deviations in the hydration abilities of the two imine groups. The right-hand side, *syn* NH group of (13) can form a strongly bent intramolecular hydrogen bond with the left-hand side (*anti* HN) nitrogen lone pair (Scheme 2). This bond reduces the hydration capacity of the molecule in the top region, but allows for favorable N…H_w_O_w_ bond formation at the right-hand side (CN = 1.3) and the (N)H…O_w_ hydration on the left-hand side (CN = 0.95). The reduced exposure of (13) to hydration, as revealed from the sum of the N_c_/O_w_ values in comparison with the double of the N_t_/O_w_ coordination number for structures (10) and (12) and with the sum of the N_t_/O_w_ values for (11) necessarily leads to the decrease of the *n*_HB_ value by 1.3–1.8 units for the *s-cis* conformer compared to that for the (10–12) *s-trans* forms.

### 2.4. Equilibration Mechanism

Equilibration of some conformers is possible both in the gas phase and in the studied solvents by rotation about the central C–C bond because the calculated barrier heights are moderate. The studied pairs meeting the conditions are (1,3), (2,4), (5,8), (6,7), (10,15), (11,13) and (12,14). The calculated ΔG^s^_tot_ values are, however, so large for (3, 4, 7, 8, 13–15) that the *s-cis* conformers could be hardly observed experimentally. The intramolecular transformation for the pair (1,2) was found as proceeding along H rotation about the C=N bond followed by N-inversion, for which the activation energy was calculated at about 111 kJ/mol in solution for (2) (Table 1).

In a dilute aqueous solution, small polar molecules could be dissolved in monomeric form at least at a large fraction. For monomers, the CC=NH *anti* to *syn* transformation must be feasible by water catalysis if the favorable hydration of structures of (1), (5), and (10–12) are considered (Table 4). More directly, conclusions from the tetrahydrate structures could be drawn (Figure 1). The water molecule w3 can donate a hydrogen and forms an N…H_w31_–O_w3_ hydrogen bond, whereas the w4 water molecule forms an N–H…O_w4_ intermolecular hydrogen bond. The *anti*/*syn* transformation would be carried out by the H_w31_ jump over to the nitrogen, whereas it releases its covalently bound proton toward O_w4_. Along a sequential mechanism, a solute-solvent ion-pair is formed, irrespective of the actual proton involved in the first proton jump. The system’s status after the first proton jump will be, however, different. If H_w32_ jumps first, the solute will be protonated facing an OH^−^ anion. If the N–H proton leaves first (less likely, since imines are not strong acids), a negatively charged solute anion and a hydroxonium cation form an ion-pair. In any case, the ion-pair could be stabilized in water for a short time until the second proton jump takes place. Now the solute is neutral, whereas there is a O_w3_H_w32_ hydroxyl anion and a HO_w4_H_2_ hydroxonium cation. The water molecules are members of a water network. Either in a concerted mechanism or through a sequential process, a double proton-relay results in the *anti* to *syn* transformation [10] and the neutralization of the hydroxyl-hydroxonium ion-pair will be reached by proton jumps along the water chain connecting O_w3_ and O_w4_. Calculated coordination and hydrogen-bond numbers are in conformity with this supposed mechanism. The outlined equilibration mechanism for the (1,2) and (5,6) pairs is a reasonable alternative of the H rotation about the C=N bond followed by an N-inversion in aqueous solution. This mechanism must fail, however, in a non-protic solvent like dichloromethane. In that solvent, equilibration may proceed along an alternative route, namely through solute association.

N–H containing molecules could produce (N–H)*_n_* rings with Nlp…H–N bonds, where lp stands for the nitrogen lone pair. Formation of a three-member ring was found experimentally even in the gas phase [41]. Through concerted proton jumps in a dimer, the *syn* conformer could come into existence. Polar solute association may be favored in a non-protic solvent, which supports the formation of intermolecular hydrogen bonds for the polar sites. Strong dimerization was found for acetic acid in chloroform [10] and for pyruvic acid in dichloromethane [1].

**Scheme 3:**
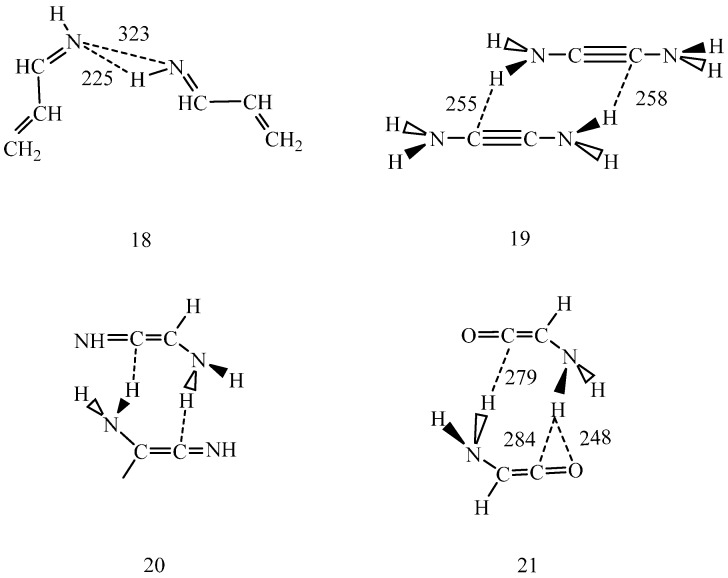
IEF-PCM/B97D/aug-cc-pvtz optimized *s-trans* 2-propene-imine dimer (18) in dichloromethane. N–H…N angle 157°, C=N…N=C torsion angle 41.2°. 2-diamino acetylene dimer, optimized in the gas phase (19). Schematic structure of the 2-amino imino-ketene dimer (20). 2-amino ketene dimer, optimized in dichloromethane (21). Dashed lines indicate selected interatomic distances in pm.

IEF-PCM/B97D/aug-cc-pvtz geometry optimization for an *s-trans* CH_2_=CH–CH=NH dimer (18) (Scheme 3) in dichloromethane predicted individually planar monomers with C=N…N=C torsion angle of 42° at 323 pm N…N distance. One intermolecular hydrogen bond was noticed with N…H distance of 225 pm and N–H…N bond angle of 157°. The other N…H distance was determined as 391 pm.

**Figure 2:**
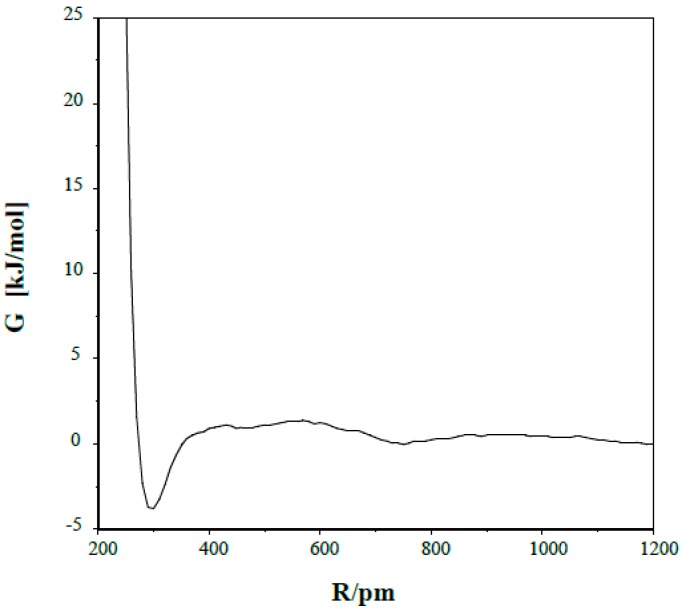
Potential of mean force for the *s-trans/anti* dimer in dichloromethane.

The potential of mean force curve (pmf) is shown in Figure 2 for a pair of the (1) species in dichloromethane. The free energy of the system (with G = 0 at N…N separation of 1200 pm) has a maximum of 1.4 ± 0.3 kJ/mol at R(N…N) = 570 pm, becomes negative below R = 350 pm and reaches its minimum of −3.8 ± 0.4 kJ/mol with N…N separation of 300 pm.

The solution concentration is about 0.12 molar corresponding to two moles of solutes in a total volume of 17 dm^3^. Assuming a small cube as its own volume for a solute, in the case of a uniform local solute density when the reference solute atoms reside at the centers of the cubes, their distances to the nearest neighbors are at about 2400 pm in a 0.12 molar solution. Thermal effects destroy this ordered arrangement, and as the calculated pmf shows, a fraction of the molecules tends to associate. The ratio of the integrals of the R^2^exp(−ΔG/*RT*) curve in the ranges of R(0, R(spec)) and R(0–2400) pm provides the fraction of the associated dimer relative to all other dimer arrangements [42]. Here, R(spec) is an appropriately chosen N…N separation, which was accepted as 570 pm in the present calculation, corresponding to the site of the top of the association barrier. G was accepted as maintaining the reference value (G = 0) at R > 1200 pm. The calculated total associated fraction was slightly more than 1%, but is much less than that in the N…N separation range of 300–320 pm required for the formation of a hydrogen-bond within the dimer.

Upon inspection of some snapshots through MC simulations, arrangements with one, primarily linear N–H…N hydrogen bond was found for the dimer at the minimum (R = 300 pm) of the pmf. This orientation is not far from the calculated optimal dimer structure and is not favorable for a double proton-relay. Although the thermal motion may lead to some bent N–H…N structure, a prerequisite for forming another hydrogen bond, the pmf does not support the concerted double proton-relay. Jump over of a single proton would result in formation of an ion-pair temporarily, whose stabilization by a moderately polar solvent like CH_2_Cl_2_ is questionable. Thus the idea of a collision-based H rotation about the C=N bond/N-inversion is left in the dicholoromethane solvent.

Exploration of the tautomerization mechanism for H_2_N–C≡C–NH_2_ (17) is also a complicated problem. Geometry optimization in the gas phase predicts linear arrangement for the heavy atoms, whereas the rotational positions of the two amino groups break any symmetry for the molecule. In fact, two optical antipodes are possible, and no easily reachable path is available for an intramolecular 1,3 hydrogen relocation, which would lead to the formation of the HN=C=CH–NH_2_ tautomer (Scheme 2).

By studying dimeric structures (19–21, Scheme 3), possibilities for their tautomerizations were investigated. As calculated at the B97D/aug-cc-pvtz level in the gas phase (for calculation details, see [10]), only about 1% of the (19) monomers form a dimer. Concerted double proton-relay is unlikely along the two weak N–H…C hydrogen bonds. A sequential mechanism may be even less likely, because the formed ion-pair could be hardly stabilized in the gas phase. Considering the above experience with the pmf calculations, even dichloromethane may not have the satisfactory stabilizing effect to facilitate either the tautomeric transformation of (19) to (20) or the transformation of the (20) dimer to yield the dimer of some of (10–12).

Structure (21) shows the intermolecular hydrogen bond pattern calculated for the optimized amino ketene dimer in dichloromethane. The structure suggests that the N–H…O hydrogen bond must be stronger than any of the N–H…C hydrogen bonds in the dimer. Accordingly, if a proton jumps to the carbonyl oxygen, then the formed structure with an OH group will definitely differ from structures (5–8). This structure may later reorganize, but this reaction path strongly suggests that tautomerization of the studied systems in the gas phase and in non-polar solvents may not proceed *via* one-step double proton-relay mechanism.

An alternative is that the kinetic control is in effect regarding the tautomerization of the present systems. The ketenes (and possibly imino-ketenes) are stable in a non-aqueous solution [12]. The calculations have found that the (9) and (16) structures correspond to local minima on the potential energy hypersurface in solution. If they were prepared in some non-aqueous solvent they may not convert to the corresponding most stable (5) and (10) structures according to the above discussion. Adding, however, even a catalytic amount of water to the solution, a provision of a proton to the carbon involved in cumulated double bonds and a drop-off of a proton at the NH_2_ site could be a feasible process. The waters are in a water network again, and the neutralization of the formed ion-pair would easily proceed through a number of proton jumps along the water bridge.

## 3. Methods and Calculations

The theoretical approaches for the present analyses follow mainly the procedures described previously [10,22,23]. Calculations were performed using the DFT/B97D method of Grimme [26] and the CCSD(T) energies were extrapolated to the complete basis set limit (CBS) by utilizing the G09 package [43] implemented at the Ohio Supercomputer Center. Both the gas-phase and solute geometries were optimized using the aug-cc-pvtz basis set [44,45,46] by the B97D method and at MP2 where applied. Local energy minima were certified with all positive vibrational frequencies calculated generally with B97D. All geometry optimizations were conducted without symmetry restrictions. In order to avoid an artificial trap into some local planarity, all torsion angles anticipated as of 0° and 180° were set to some values deviating from the target value by 1°–2° in the starting geometries. Optimization could then reach an optimal, largely different value or would return to about 0° and 180°. Perfect local planarity is not expected from such starting geometries through symmetry unrestricted optimizations, thus predicted optimal torsion angles, when the standard optimization criteria were used in Gaussian 09, could slightly differ from 0° and 180°. No significance was paid to deviations up to 0.2°. With larger values two antipodes have to be considered, whose mixing would result in −*RT* ln 2 = −1.717 kJ/mol entropy change; see detailed geometry parameters in Supplementary Table S1. For solutions, geometries of the tautomers and conformers (Scheme 1 and Scheme 2) were optimized by means of the IEF-PCM approach (integral equation formalism of the polarizable continuum method) [47,48].

The relative free energy for a pair of tautomers/conformers in solution was calculated as:

ΔG^s^_tot_ = (ΔE^s^_int_ + ΔG^s^_th_) + (ΔE_elst_ + ΔG_drc_) ≡ (ΔE^s^_int_ + ΔG_th_) + ΔG_solv_/PCM (1)

where the terms ΔE^s^_int_ + ΔG^s^_th_ stand for the relative internal energy and the thermal corrections, respectively. The final ΔE^s^_int_ values were accepted on the basis of B97D/aug-cc-pvqz single-point calculations in DFT calculations. The thermal corrections were calculated in the rigid rotor-harmonic oscillator approach [49]. ΔE_elst_ and ΔG_drc_ account for the relative solute-solvent electrostatic interaction energy and the relative dispersion-repulsion-cavitation free energy, respectively. The dielectric constants of the dichloromethane and water solvents were set to 8.93 and 78.39, respectively, and scaled Bondi radii [31,50] were accepted for carving the cavity for polar solute atoms.

Relative CCSD(T) in-solution internal energies at the complete basis set limit (CBS) were calculated by using the formula of Hobza [51]:

ΔE^CCSD(T)^_CBS_ = ΔE^MP2^_CBS_ + (ΔE^CCSD(T)^ − ΔE^MP2^)_aug-cc-pvdz_ (2)

where ΔE^MP2^_CBS_ was obtained upon the extrapolation formula of Helgaker, *et al.* [52] for the complete basis set limit MP2 energy as:

E^MP2^_CBS_ = E(X) − A/X^3^ (3)

“A” and then E^MP2^_CBS_ in Equation (3) were obtained by single-point calculations using the aug-cc-pvdz (X = 2) and aug-cc-pvtz (X = 3) basis sets at the aug-cc-pvtz optimized geometries. Relative solvation free energies were obtained at the MP2/aug-cc-pvtz level after IEF-PCM optimization.

Monte Carlo (MC) simulations in dichloromethane and water solvents were performed in the NpT (isobaric-isothermal) ensemble at p = 1 atm and T = 298 K [53,54,55]. Calculations were carried out by using the BOSS 4.8 package [56]. Atomic interaction energies were calculated by means of the all-atom OPLS-AA 12-6-1 pair-potential [57,58] with the 12-6 Lennard-Jones parameters taken from the program’s library. By means of the CHELPG procedure [32], the atomic charges for the solutes were fitted to the IEF-PCM/B97D/aug-cc-pvqz molecular electrostatic potential generated by the polarized solute in the continuum solvent.

The solutes were immersed in a box of 506 TIP4P water molecules [37] or in a box of 263 dichloromethane molecules, using the three-point OPLS CH_2_Cl_2_ model [59]. All cutoffs were set to 12 Å, preferential sampling and periodic boundary conditions were applied and the long-range electrostatic effects were taken into account by means of the Ewald summation [60,61]. The relative solvation free energy, ΔG_solv_/MC for a pair of solutes was calculated by means of the free energy perturbation method (FEP) [38,39]. The solution was equilibrated by allowing the development of 7.5 M configurations, and another 7.5 M configurations were utilized for averaging the relative solvation free energy. Solution structure characteristics were obtained by integration of radial distribution functions [40] until their first minima, and by integration of solute-solvent pair-energy distribution functions (pedfs). The integration of a pedf until its first minimum provides the number of solvent molecules strongly bound to the solute. Possible dimerization of the all-trans CH_2_=CH–CH=NH species (1) in dichloromethane was followed by calculating the potential of mean force curve. A model solution-box was composed of 259 dichloromethane and two solute molecules, and FEP/MC interaction potential and simulation parameters at T = 298 K were accepted as for the single-solute models above. The change of the free energy of the system was calculated in the R(N…N) separation range of 250–1200 pm. The incremental ΔG_i(solv)_ changes were calculated at R ± 10 pm with ΔR steps of 20 pm. The N…N separation was fixed at each selected reference point. The solutes could move only in tandem, but independent rotations of the component solutes about randomly selected axes through a randomly selected N atom were allowed. More details were provided in former papers [1,10] and references therein.

## 4. Conclusions

Conformational/tautomeric isomerizations for C_3_H_5_N, C_2_H_3_NO, and C_2_H_4_N_2_ molecules have been studied in the gas phase, in dichloromethane, and in aqueous solutions. The most stable studied species within an indicated composition group adopts a double bond-single bond-double bond structure, called DSD, in the form of X=CH–CH=Y. This paper is a continuation of the study for DSD structures with all combinations of CH_2_ and O for X and Y [1]. DSD molecules are subject to *s-trans*/*s-cis* conformational variety with XCCY torsional angles of 180° and near 0°, respectively. For the present most stable structures with X=CH_2_, O, NH and Y=NH, the *s-trans* conformation is preferred in the gas phase. The preference is based on the internal free energy calculated at the B97D/aug-cc-pvqz and the CCSD(T)_CBS_ levels. The two methods predict in accord that the internal free energy is generally considerably lower for the *s-trans*, CCNH *anti* species than that for any *s-cis* form. Possibility for the existence of an intramolecular hydrogen bond and its structure-stabilization effect was discussed for the *s-cis* CCNH *syn* forms.

Transition state barriers for rotation about the central C–C bonds are of about 29–36 kJ/mol, which can be overridden upon collision. For the C–C=N–H *anti* to *syn* conformational equilibration, the calculated route toward the transitions state is a structural change along H rotation about the C=N bond followed by N-inversion. Reaching TS requires 113–123 kJ/mol activation energy in the gas phase (about 110 kJ/mol in solution) and more than 100 kJ/mol activation free energy. Nonetheless, experiments showed that both *s-trans/anti* and *syn* forms exist in the gas phase at T = 673 K [29].

Relative in-solution free energies were calculated in the IEF-PCM, polarizable continuum dielectric solvent approximation, and relative solvation free energies were estimated for a number of transformations by means of the free energy perturbation/Monte Carlo simulations utilizing explicit solvent models. The *s-trans/anti* form, due to its much lower internal free energy than that for any *s-cis*, remains prevalent, almost exclusively, in condensed phase. In many cases, however, ΔG_solv_ is negative for an *s-cis* form, but these values are never enough to reverse the conformational predominance. The FEP/MC relative solvation free energy is less negative by 0.6–9.3 kJ/mol then the corresponding IEF-PCM ΔG_solv_ value with generally equal sign for the two terms. This means that ΔG^s^_tot_ becomes more positive when ΔG_solv_ from FEP/MC rather than from IEF-PCM is considered and the relative stability of the conformer/tautomer preferred by IEF-PCM further increases. Even the limited number of FEP/MC calculations pointed out significant deviations from the IEF-PCM predicted ΔG_solv_, mainly in aqueous solution. The difference may be attributed to the different appreciation of the solute-water hydrogen bonds by the two methods. In the absence of experimental data in the present case, one cannot decide, which method of ΔG_solv_ estimation would result in quantitatively more correct ΔG^s^_tot_. Former studies [16,24] proved that both methods could predict solvent compositions in fairly good accord with experimental data at least for the major components (e.g., *gauche* or *trans*), thus the favorable method for the calculation may also depend on the system itself.

The effect of the first-shell water on the relative conformer energies have been studied in the supermolecule/continuum solvent approximation. In the *s-cis* solute conformations of the tetrahydrate supermolecules, water-water hydrogen bonds are formed, which make an additional stabilization for the tetrahydate. An analysis pointed out that this energy result is a consequence of the consideration of a limited number of waters. By application of reasonable corrections, ΔE^s^_int_ + ΔG_solv_ energies were obtained close to the values, which were calculated for pure solutes in water by IEF-PCM. Explicit water molecules in the supermolecule do not form water-water bond(s) in the transition state through the rotation about the central C–C bond and the decrease of barrier is small compared to the calculated corresponding value for a pure solute placed in the solvent cavity.

For the less stable species, the tautomerization routes of a ketene or an imino-ketene form to a DSD structure were followed and the possible role of the solvents in these processes was discussed. A proposed mechanism utilizing the water-catalyzed double proton-relay in CCNH *anti* to *syn* transformations was outlined by considering the intermolecular hydrogen bond structure in tetrahydates. Solution structure characteristics, solute-solvent hydrogen-bond patterns derived on the basis of Monte Carlo simulations are in conformity with the proposed mechanism. No clear answer could be given, however, concerning the possible catalytic effect of dichloromethane through proton transfers. Solute dimerization in this solvent does not facilitate a double proton-relay. Even the possibility was raised that the kinetic control regulates the tautomerism in an absolute water-free non-protic solvent and maintains species of high relative free energy.

Changes of the solute atom coordination numbers by the first-hydration-shell water atoms upon conformational/tautomeric transformations were analyzed for all composition groups. Hydration of the carbonyl oxygen in O=CH–CH=NH is less favorable in its *s-cis* conformation with an intramolecular hydrogen bond (7, CCNH *syn*) and in the amino ketene tautomer (9) than in conformations with CCNH *anti* moieties (5,8). The NH groups are differently open to hydration for the NH=CH–CH=NH conformers. There is an intramolecular hydrogen bond in the *s-cis*/CCNH/*syn* form (13), which hinders remarkably the formations of N…H_w_–O_w_ and N–H…O_w_ hydrogen bonds in the indicated domain.

For systems including nearby, possibly interacting hydrogen-bonding sites, special molecular mechanics parameterization is needed. To this aim, proper determination of the molecular geometries and relative free energies for conformers/tautomers is required in condensed phase. Application of high-level theoretical methods is necessary for calculating correct relative internal free energies and good estimation of the relative solvation free energy is essential, as well. Regarding this latter issue, the question is whether ΔG_solv_ should be calculated in a polarizable continuum solvent approximation or by utilizing the free energy perturbation method applied for solutions with explicit solvent models. Although the problem is known for a long time, its reassuring resolution has not been possible mainly due to the lack of a large number and reliable experimental studies determining in-solution equilibrium compositions including fractions for minor components, as well.

## Supplementary Materials

Supplementary materials can be found at http://www.mdpi.com/1422-0067/16/05/10767/s1.

It is available for the major optimized geometric parameters and dipole moments in the gas phase and in the two solvents (Table S1 and Figure S1a–d).
