# Supplementary material for: Theoretical in-Solution Conformational/Tautomeric Analyses for Chain Systems with Conjugated Double Bonds Involving Nitrogen(s)
Source: Int J Mol Sci. 2015 May 13;16(5):10767–96. doi: 10.3390/ijms160510767 (PMC4463675; doi:10.3390/ijms160510767)
Supplement: Supplementary file 1 [file ijms-16-10767-s001.pdf]

Supplementary Information

Table S1. Major optimized geometric parameters using the aug-cc-pvtz basis set. Data are rounded to the last decimal [a]. (Experimental values in parentheses).

Structures in Schemes	B97D [b]			MP2 [c]		
	Gas	CH_2Cl_2	Water	Gas	CH_2Cl_2	Water
CH2=CH–CH=NH						
C=C (1)	134.1	134.1	134.1	133.9 (133.6)	133.9	133.9
(2)	134.1	134.1	134.1	133.9	133.9	133.9
(3)	134.1	134.0	134.0	133.9	133.8	133.8
(4)	133.9	133.9	133.9			133.8
C–C (1)	146.1	145.8	145.8	145.8 (145.4)	145.6	145.6
(2)	146.6	146.2	146.1	146.2	145.9	145.8
(3)	147.7	147.5	147.3	147.2	147.1	147.1
(4)	148.1	147.8	147.7			147.0
C=N (1)	128.2	128.4	128.5	128.4 (127.4)	128.5	128.5
(2)	128.1	128.4	128.5	128.3	128.5	128.5
(3)	128.1	128.3	128.3	128.3	128.3	128.3
(4)	127.9	128.2	128.3			128.3
N–H (1)	102.3	102.1	102.2	102.0 (101.4)	101.9	101.9
(2)	102.8	102.6	102.6	102.3	102.2	102.3
(3)	102.2	102.1	102.1	101.9	101.8	101.8
(4)	102.8	102.6	102.6			102.3
CCC (1)	122.4	121.8	121.6	121.9 (122.9)	121.3	121.2
(2)	122.7	122.2	122.0	122.1	121.4	121.4
(3)	124.5	124.8	124.7	123.1	123.6	123.6
(4)	124.6	124.6	124.6			122.8
CCN (1)	121.4	122.0	122.0	120.7 (121.5)	121.3	121.4
(2)	127.4	126.8	126.6	126.9	126.3	126.1
(3)	123.0	123.5	123.5	121.8	122.3	122.4
(4)	128.5	128.3	128.3			126.5
CNH (1)	110.5	109.9	109.8	110.1 (111.7)	109.6	109.4
(2)	110.3	109.9	109.8	109.7	109.4	109.2
(3)	110.5	109.8	109.7	109.9	109.3	109.2
(4)	110.5	110.4	110.4			109.3
CCCN (1)	180.0	180.0	180.0	180.0	180.0	180.0
(2)	180.0	180.0	180.0	180.0	180.0	180.0
(3)	0.0	5.7	13.1	0.0	0.2	0.2
(4)	22.5	17.6	15.9			30.6
CCNH (1)	180.0	180.0	180.0	180.0	180.0	180.0
(2)	0.0	0.0	0.0	0.0	0.0	0.0
(3)	180.0	180.4	180.6	180.0	180.0	180.0
(4)	1.4	0.9	0.8			1.7
**Dipole moments**						
(1)	2.08	2.84	3.02	2.13 (2.01)	2.82	2.98
(2)	2.65	3.46	3.63	2.67 (2.51)	3.39	3.53
(3)	1.62	2.33	2.43	1.70	2.30	2.45
(4)	2.51	3.26	3.41			3.31

Table S1. *Cont.*

Structures in Schemes	B97D [b]			MP2 [c]		
	Gas	CH_2Cl_2	Water	Gas	CH_2Cl_2	Water
**TS (1 to 2, CCNH Torsion)**						
C=C	134.0	134.0	134.0	133.9		
C–C	148.1	147.9	147.8	147.6		
C=N	124.5	124.7	124.8	124.5		
N–H	99.0	99.2	99.2	99.0		
CCC	121.6	121.1	121.1	120.9		
CCN	125.6	125.7	125.6	125.3		
CNH	179.3	179.6	179.7	179.2		
CCCN	180.0	180.2	180.2	180.0		
CCNH	124.9	125.3	125.4	125.4		
**Dipole Moments**						
TS(1 to 2)	0.57	0.82	0.89	0.54		
**TS (1 to 3, CCCN Torsion)**						
C=C	133.3	133.3	133.3	133.4		
C–C	149.1	148.9	148.8	148.3		
C=N	127.5	127.6	127.7	127.9		
N–H	102.5	102.3	102.3	102.1		
CCC	123.8	123.7	123.6	122.8		
CCN	121.6	121.9	121.9	120.7		
CNH	110.2	109.9	109.9	109.7		
CCCN	95.4	96.0	96.3	96.1		
CCNH	179.7	179.5	179.5	179.9		
**Dipole Moments**						
TS (1 to 3)	1.88	2.51	2.67	1.96		
O=CH–CH=NH						
O=C(5)	121.3	121.7	121.7	121.7	121.9	121.9
(6)	121.0	121.4	121.5			
(7)	121.2	121.5	121.5	121.6	121.8	121.8
(8)	120.8	121.4	121.5	121.2	121.6	121.7
(9)	117.2	117.5 [b]	117.4	117.1	117.1	117.1
C–C(5)	150.2	149.8	149.7	149.0	148.7	148.6
(6)	150.5	150.3	150.2			
(7)	152.1	152.0	152.0	150.6	150.5	150.5
(8)	152.0	151.6	151.5	150.6	150.2	150.1
(9)	132.4	132.2 [b]	132.1	132.6	132.4	132.3
C–N(5)	127.6	127.6	127.6	127.9	127.9	127.9
(6)	127.6	127.6	127.6			
(7)	127.3	127.3	127.3	127.6	127.7	127.7
(8)	127.3	127.4	127.4	127.7	127.7	127.7
(9)	144.6	145.0 [b]	144.8	143.4	143.7	143.8
N–H (5)	102.5	102.3	102.3	102.1	102.0	102.0
(6)	103.1	102.9	102.8			
(7)	103.1	102.9	102.9	102.8	102.6	102.6

Table S1. *Cont.*

Structures in Schemes	B97D [b]			MP2 [c]		
	Gas	CH_2Cl_2	Water	Gas	CH_2Cl_2	Water
(8)	102.5	102.3	102.3	102.1	102.0	101.9
(9)	101.5	101.6 [b]	101.5	101.0		
OCC (5)	122.7	122.9	122.9	122.5	122.6	122.7
(6)	124.2	123.6	123.5			
(7)	122.9	122.5	122.4	121.8	121.4	121.3
(8)	125.1	124.5	124.4	124.0	123.4	123.3
(9)	177.3	177.4 [b]	178.0	178.8	179.0	179.0
CCN (5)	118.4	118.0	117.9	117.6	117.2	117.2
(6)	123.8	123.2	123.1			
(7)	124.5	124.0	123.9	122.9	122.4	122.2
(8)	121.2	120.9	120.8	120.1	119.8	119.8
(9)	120.1	120.1 [b]	120.5	121.8	121.8	121.7
CNH (5)	111.2	110.9	110.8	110.8	110.5	110.4
(6)	110.5	110.5	110.5			
(7)	109.4	109.6	109.6	107.9	107.9	107.9
(8)	110.7	110.2	110.2	110.0	109.6	109.5
(9)	112.8	111.9 [b]	111.6	112.9		
OCCN (5)	180.0	179.8	179.8	180.0	179.9	179.9
(6)	180.0	180.0	180.0			
(7)	0.0	0.0	0.0	0.0	0.0	0.0
(8)	0.4	6.2	6.6	0.4	11.1	10.4
(9)	117.4	117.6 [b]	118.5	120.6	120.9	120.8
CCNH (5)	180.0	180.0	180.0	180.0	180.0	180.0
(6)	0.0	0.0	0.0			
(7)	0.0	0.0	0.0	0.0	0.0	0.0
(8)	180.0	179.7	179.3	180.0	179.5	179.5
(9)	61.9	60.8	64.5	62.4	64.2	64.6
	−61.9	−60.3	−55.9	−62.2	−57.2	−55.8
**Dipole Moments**						
(5)	2.28	2.84	2.95	2.25	2.76	2.86
(6)	1.40	1.87	1.97			
(7)	2.03	2.69	2.84	2.34	3.03	3.17
(8)	3.88	5.02	5.28	4.07	5.18	5.44
(9)	0.96	1.13	1.16	0.74	0.86	0.88
**TS (6 to 7, CCCN Torsion)**						
O=C	121.1	121.5	121.5	121.6		
C–C	151.9	151.5	151.4	150.2		
C=N	127.3	127.4	127.4	127.8		
N–H	102.8	102.7	102.6	102.4		
OCC	122.9	122.4	122.2	122.5		
CCN	124.3	123.9	123.9	124.1		
CNH	111.5	111.6	111.6	110.5		
OCCN	87.3	88.2	88.4	89.2		
CCNH	0.2	0.5	0.5	0.2		

Table S1. *Cont.*

Structures in Schemes	B97D [b]			MP2 [c]		
	Gas	CH_2Cl_2	Water	Gas	CH_2Cl_2	Water
**Dipole Moments**						
TS (6 to 7)	1.92	2.49	2.61	2.05		
HN=CH–CH=NH						
H–N (10)	102.4	102.3	102.2	102.0	102.0	101.9
(11)	102.4	102.3	102.2	102.0	102.0	101.9
(12)	102.8	102.8 [b]	102.8 [b]	102.4	102.3	102.3
(13)	102.2	102.2 [b]	102.2 [b]	101.9	101.8	101.8
(14)	103.0 [b]	102.8	102.8		102.5	102.4
(15)	102.4 [b]	102.3	102.2		102.0	102.0
(16)	102.6 [b]	102.7 [b]	102.5	102.1		
N=C (10)	127.9	127.9	127.9	128.1	128.1	128.1
(11)	127.7	127.8	127.8	128.0	128.0	128.0
(12)	127.7	127.8 [b]	127.6 [b]	128.0	128.0	128.0
(13)	127.6	127.6 [b]	127.6 [b]	127.9	127.9	127.9
(14)	127.5 [b]	127.6	127.6		127.9	127.9
(15)	127.5 [b]	127.7	127.7		127.9	128.0
(16)	123.7 [b]	124.0 [b]	123.9	123.9		
C–C (10)	147.8	147.6	147.6	147.0	147.0	146.9
(11)	148.2	148.1	148.1	147.4	147.3	147.3
(12)	148.6	148.6 [b]	148.6 [b]	147.7	147.7	147.6
(13)	150.1	150.2 [b]	150.2 [b]	149.1	149.1	149.0
(14)	150.6 [b]	150.3	150.4		149.1	149.0
(15)	149.4 [b]	149.3	149.3		148.3	148.3
(16)	132.1 [b]	132.0 [b]	131.9	131.9		
C–N (10)	127.9	127.9	127.9	128.1	128.1	128.1
(11)	127.8	127.9	127.9	128.1	128.1	128.1
(12)	127.7	127.8 [b]	127.8 [b]	128.0	128.0	128.0
(13)	127.4	127.7 [b]	127.7 [b]	127.8	127.9	127.9
(14)	127.5 [b]	127.6	127.6		127.9	127.9
(15)	127.5 [b]	127.7	127.7		127.9	128.0
(16)	142.9 [b]	142.9 [b]	142.7	142.6		
N–H (10)	102.4	102.3	102.2	102.0	102.0	101.9
(11)	102.9	102.7	102.7	102.4	102.3	102.3
(12)	102.8	102.8 [b]	102.8 [b]	102.4	102.3	102.3
(13)	102.9	102.9 [b]	102.9 [b]	102.7	102.7	102.6
(14)	103.0 [b]	102.8	102.8		102.5	102.4
(15)	102.4 [b]	102.3	102.2		102.2	102.2
(16)	101.5 [b]	101.5 [b]	101.4	101.2		
(16)	101.7 [b]	101.7 [b]	101.6	101.3		
HNC (10)	110.7	110.4	110.3	110.3	110.0	110.0
(11)	110.0	110.5	110.4	110.5	110.1	110.0
(12)	110.5	110.5 [b]	110.5 [b]	109.9	109.7	109.7
(13)	111.2	110.8 [b]	110.7 [b]	110.8	110.2	110.1
(14)	111.3 [b]	111.3	111.5		110.3	110.3

Table S1. *Cont.*

Structures in Schemes	B97D [b]			MP2 [c]		
	Gas	CH_2Cl_2	Water	Gas	CH_2Cl_2	Water
(15)	110.2 [b]	109.8	109.7		109.3	109.2
(16)	114.5 [b]	114.2 [b]	114.0	113.7		
NCC (10)	119.6	119.9	119.9	119.0	119.3	119.3
(11)	120.5	120.2	120.2	119.5	119.3	119.3
(12)	126.2	125.5 [b]	125.4 [b]	125.6	124.9	124.7
(13)	120.8	120.9 [b]	120.9 [b]	119.3	119.2	119.1
(14)	127.6 [b]	126.9	127.1		125.8	125.7
(15)	122.6 [b]	122.7	122.7		121.2	121.2
(16)	168.5 [b]	169.1 [b]	169.7	170.0		
CCN (10)	119.6	119.9	119.9	119.0	119.3	119.3
(11)	125.4	125.1	125.1	125.1	124.7	124.7
(12)	126.2	125.5 [b]	125.4 [b]	125.6	124.9	124.7
(13)	126.4	126.2 [b]	126.2 [b]	124.4	123.9	123.7
(14)	127.6 [b]	126.9	127.1		125.8	125.7
(15)	122.6 [b]	122.7	122.7		121.2	121.2
(16)	125.4 [b]	125.9 [b]	126.0	124.6		
CNH (10)	110.7	110.4	110.3	110.3	110.0	110.0
(11)	110.5	110.3	110.3	109.8	109.7	109.6
(12)	110.5	110.5 [b]	110.5 [b]	109.8	109.7	109.7
(13)	109.5	109.6 [b]	109.7 [b]	107.8	107.6	107.5
(14)	111.3 [b]	111.3	111.5		110.3	110.3
(15)	110.2 [b]	109.8	109.7		109.3	109.2
(16)	112.0 [b]	112.1 [b]	112.0	111.4		
(16)	111.9 [b]	111.1 [b]	111.0	111.6		
HNCC (10)	180.0	180.0	180.0	180.0	179.9	180.0
(11)	180.0	180.0	180.0	180.0	180.0	180.0
(12)	0.0	0.0 [b]	0.0 [b]	0.0	0.0	0.0
(13)	180.0	180.0 [b]	180.0 [b]	180.1	180.1	180.1
(14)	0.5 [b]	0.6	0.5		0.7	0.7
(15)	179.8 [b]	179.8	179.8		180.0	180.0
(16)	126.1 [b]	125.1 [b]	124.3	123.7		
NCCN (10)	180.0	180.0	180.0	180.0	180.0	180.0
(11)	180.0	180.0	180.0	180.0	180.0	180.0
(12)	180.0	179.9 [b]	179.9 [b]	180.0	180.0	180.1
(13)	0.0	0.2 [b]	0.2 [b]	0.1	0.1	0.1
(14)	14.0 [b]	17.6	14.9		21.4	22.2
(15)	26.8 [b]	22.7	18.8		29.8	27.7
(16)	123.1 [b]	124.1 [b]	124.9	122.7		
CCNH (10)	180.0	180.0	180.0	180.0	180.1	180.0
(11)	0.0	0.0	0.0	0.0	0.0	0.0
(12)	0.0	0.0 [b]	0.0 [b]	0.0	0.0	0.0
(13)	0.0	0.0 [b]	0.0 [b]	0.0	0.0	0.0
(14)	0.5 [b]	0.6	0.5		0.7	0.7
(15)	179.8 [b]	179.8	179.8		180.0	180.0

Table S1. *Cont.*

Structures in Schemes	B97D [b]			MP2 [c]		
	Gas	CH_2Cl_2	Water	Gas	CH_2Cl_2	Water
(16)	1.7 [b]	7.0 [b]	7.3	−7.0		
(16)	120.4 [b]	127.9 [b]	127.9 [b]	114.0		
**Diamino Acetylene (17)**						
H–N	101.3	101.4 [b]	101.3			
N–C	135.1	135.4 [b]	135.3			
C≡C	121.5	121.8 [b]	121.7			
HNC(ave)	116.6	116.0 [b]	116.1 [b]			
H_1NNH_3	130.8	133.6 [b]	132.8			
H_1NNH_4	−90.8	−90.6 [b]	−90.4			
H_2NNH_3	−90.8	−90.6 [b]	−90.1			
H_2NNH_4	47.7	45.2 [b]	44.7			
**Dipole Moments**						
(10)	0.0	0.0	0.0	0.0	0.0	0.0
(11)	2.68	3.41	3.58	2.83	3.56	3.72
(12)	0.0	0.0	0.0	0.0	0.0	0.0
(13)	3.11	4.03	4.22	3.42	4.36	4.88
(14)	0.67	0.90	0.91		1.09	1.14
(15)	3.31	4.46	4.76		4.60	4.89
(16)	2.37	3.17	3.35	2.38		
(17)	1.19	1.62	1.68			
**TS (11 to 13, CCCN Torsion)**						
H–N	102.4	102.3	102.3	102.1		
N=C	27.3	127.4	127.4	127.8		
C–C	150.4	150.1	150.1	149.1		
C=N	127.3	127.5	127.5	127.8		
N–H	102.8	102.6	102.6	102.3		
HNC	110.7	110.2	110.1	110.1		
NCC	120.7	120.8	120.7	119.7		
CCN	125.3	125.1	125.0	124.8		
CNH	110.9	110.8	110.8	109.9		
HNCC	−178.2	−178.5	−178.5	−178.5		
NCCN	92.7	93.2	93.4	93.6		
CCNH	−1.1	−0.7	−0.5	−0.6		
**Dipole Moments**						
TS (12 to 13)	2.90	3.70	3.89	3.11		
**TS (10 to 15, CCCN Torsion)**						
H–N	102.4	102.4	102.1			
N=C	127.2	127.4	127.7			
C–C	150.2	150.2	148.9			
C=N	127.2	127.4	127.7			
N–H	102.4	102.4	102.1			
HNC	110.5	110.2	110.0			
NCC	120.8	120.6	119.8			

Table S1. *Cont.*

Structures in Schemes	B97D [b]			MP2 [c]		
	Gas	CH_2Cl_2	Water	Gas	CH_2Cl_2	Water
CCN	120.8	120.6	119.8			
CNH	110.5	110.2	110.0			
HNCC	179.6	179.7	180.0			
NCCN	86.3	93.5	83.6			
CCNH	179.6	179.7	180.0			
**TS (10 to 15, CCCN Torsion)**						
H–N	102.4	102.4	102.1			
N=C	127.2	127.4	127.7			
C–C	150.2	150.2	148.9			
C=N	127.2	127.4	127.7			
N–H	102.4	102.4	102.1			
HNC	110.5	110.2	110.0			
NCC	120.8	120.6	119.8			
CCN	120.8	120.6	119.8			
CNH	110.5	110.2	110.0			
HNCC	179.6	179.7	180.0			
NCCN	86.3	93.5	83.6			
CCNH	179.6	179.7	180.0			
**Dipole Moments**						
TS (10 to 15)	2.67	3.48	2.84			

[a] Distances in pm, angles in deg; [b] Where superscript "b" was indicated for B97D parameters, the values were obtained through Berny optimization (numerical second derivative method). All positive frequencies were achieved where were calculated. B97D parameters without superscript were obtained from optimizations using analytical second derivatives. Application of this latter method was needed in order to obtain all positive frequencies; [c] All MP2 optimizations with the Berny method.

(**a**)

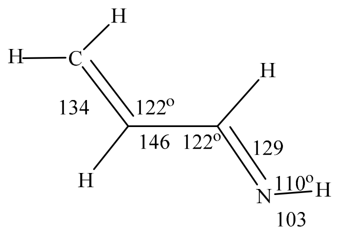

1 tA (sym C_S)

CCCN=180°

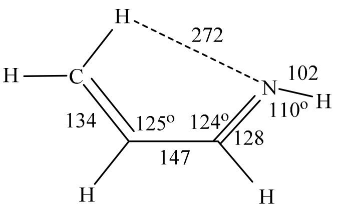

3 cA (sym C_1)

CCCN=13°

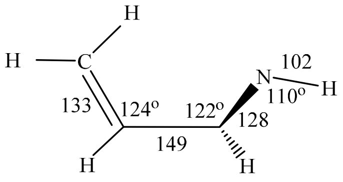

TS (sym C_1)

CCCN=96°

(**b**)

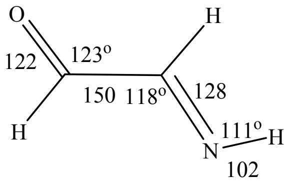

5 tA (sym C_S)

OCCN=180°

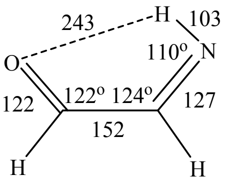

7 cS (sym C_S)

OCCN=0°

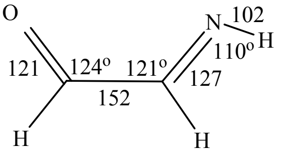

8 cA (sym C_1)

OCCN=7°

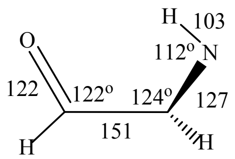

TS (sym C_1)

OCCN=88° CCNH=1°

Figure S1. *Cont.*

(c)

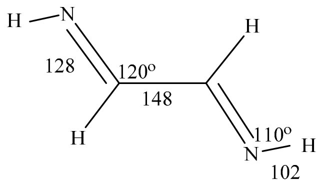

10 tAA (sym C_{2h})

NCCN=180°

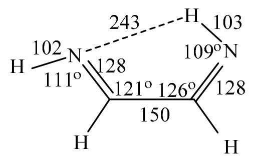

13 cAS (sym C_S)

NCCN=0°

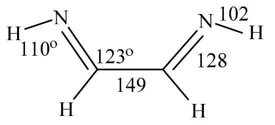

15 cAA (sym C_2)

NCCN=19°

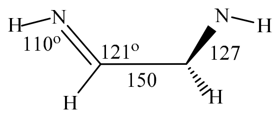

TS (sym C_2)

NCCN=94°

(d)

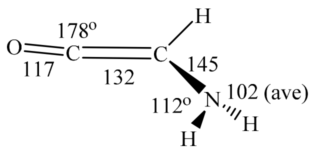

9 (sym C_1)

OCCN=119°, CCNH=65, -56°

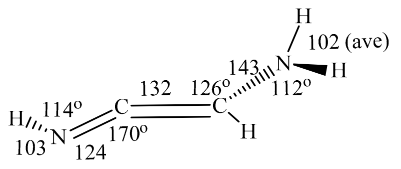

16 (sym C_1)

HNCC=124°, NCCN=125°, CCNH=7°, 128°

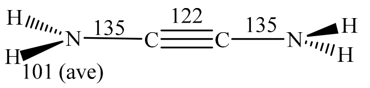

17 (sym C_1)

HNNH=133°, -90°, -90°, 45°

Figure S1. (a–d) Structure numbers from Schemes. IEP-PCM/B97D/aug-cc-pvtz optimized structural parameters in aqueous solution for species primarily discussed in the paper. Values appear at three digits. Four-digit internal coordinates are provided in the Table S1 for all optimized structures in different environments as calculated at the B97D and MP2 levels. The distances are provided in pm (although pm specification is not indicated because of the crowded drawings). Bond angle values are followed by the degree symbol. HNCC torsion angles are 180° or 0°, otherwise shown.
